# Liposomal co-delivery of β-carotene and doxorubicin for enhanced colorectal-cancer therapy

**DOI:** 10.1038/s41598-025-27935-5

**Published:** 2025-12-18

**Authors:** Diana M. Alhazzaa, Medhat W. Shafaa, Seifeldin Elabed, Ahmed M. Shafaa, Mohamed M. Omran

**Affiliations:** 1 Biochemistry Division, Chemistry Department, Faculty of Science, Helwan University, Cairo, Egypt; 2 Medical Biophysics Division, Physics Department, Faculty of Science, Helwan University, Cairo, Egypt; 3 Biotechnology and Genetic Engineering Department, Faculty of Science, Helwan National University, Cairo, Egypt; 4 Biochemistry Department, Faculty of Agriculture, Cairo University, Cairo, Egypt

**Keywords:** Biochemistry, Biotechnology, Cancer, Drug discovery, Nanoscience and technology

## Abstract

Colorectal cancer (CRC) remains a major cause of cancer-related mortality, motivating delivery systems that retain antitumor activity while limiting off-target toxicity. We engineered neutral multilamellar soy-lecithin liposomes co-encapsulating β-carotene (BC) and doxorubicin (DOX). Thin-film hydration produced vesicles with a DLS Z-average of 122.4 ± 83.4 nm (PDI = 0.534) and ζ-potential of − 25.6 ± 8.0 mV, and a TEM diameter of 184 ± 34 nm, with > 90% entrapment efficiency for both cargos. In HCT-116 cells the formulation preserved DOX potency (IC₅₀, µg mL⁻^1^, mean ± SD: free DOX 610.9 ± 11.4; DOX-Lipo 614.0 ± 1.8; BC–DOX-Lipo 618.9 ± 5.8) and remained ~ 1.5-fold more active than BC alone (free BC 960.6 ± 13.6; BC-Lipo 951.8 ± 18.8). Annexin V/PI flow cytometry showed that BC–DOX-Lipo achieved the highest total apoptosis (44.51 ± 4.45%) and deep G₀/G₁ arrest (92.06 ± 9.2%), reducing S-phase to 6.37 ± 0.60% (p = 0.0186 vs DOX-Lipo). Alkaline comet analysis indicated that co-delivery modestly attenuated DOX-associated DNA fragmentation (tailed cells 11.43 ± 0.45% for BC–DOX-Lipo vs 16.27 ± 0.25% for DOX-Lipo, p < 0.001), consistent with BC’s redox buffering. Differential scanning calorimetry and FTIR supported drug–bilayer interactions: BC slightly increased Tm and acyl-chain order, whereas DOX disrupted cooperativity; co-loading shifted T_m_ to 37.84 °C and broadened the transition, indicating a more fluid bilayer at 37 °C. In-silico ADMET profiling (contextual, not in-vivo PK) highlighted superior oral absorption and BBB penetration for BC and negligible oral uptake for DOX; docking predicted higher DOX affinities across Bcl-2, β-catenin, P-glycoprotein, and topoisomerase II (e.g., − 9.06 to − 9.30 kcal mol⁻^1^) relative to BC (≈ − 7.6 to − 7.8 kcal mol⁻^1^). Overall, liposomal co-delivery maintains DOX cytotoxicity while strengthening G₁/S checkpoint blockade and increasing programmed cell death, with partial moderation of DNA fragmentation. These in-vitro data motivate stability optimization and in-vivo evaluation in CRC models.

## Introduction

Cancer, a complex disease marked by uncontrolled cell growth and spread, remains a global health crisis and the second leading cause of death after cardiovascular diseases^1^. Colorectal cancer (CRC), the third most common malignancy, causes approximately 500 000 deaths annually, underscoring the urgent need for safer, more effective therapies^2^. Conventional treatments—surgery, radiation, chemotherapy, hormone therapy, immunotherapy, and targeted therapy—aim to eliminate tumours but often damage healthy tissues, leading to dose-limiting toxicities and long-term side effects^3,4^.

Nanotechnology has revolutionised oncology by enabling targeted drug delivery, precise diagnostics, and controlled-release systems^2^. Various nanocarriers—including liposomes, micelles, nanogels, dendrimers, and vesicles—have been designed to enhance drug efficacy, reduce off-target effects, and overcome physiological barriers^3,5^. Lipid-based carriers are especially promising owing to their biocompatibility, biodegradability, and ability to evade rapid clearance by the reticulo-endothelial system (RES), thereby prolonging circulation and enhancing tumour accumulation via the enhanced permeability and retention (EPR) effect^2,5^.

Liposomes—spherical vesicles comprising one or more phospholipid bilayers enclosing an aqueous core—are exemplary lipid-based delivery systems^6^. Their amphiphilic nature enables simultaneous loading of hydrophilic and hydrophobic agents, improving solubility and stability^6^. Lecithin, particularly phosphatidylcholine, is frequently used in liposome synthesis for its surfactant properties, membrane compatibility, and intravenous safety profile^6,7^. Studies show that liposomal encapsulation of cytotoxic agents such as cyclophosphamide and doxorubicin significantly reduces systemic toxicity, including myelosuppression and cardiotoxicity, compared with free-drug formulations^7,8^.

**Fig. 1:**
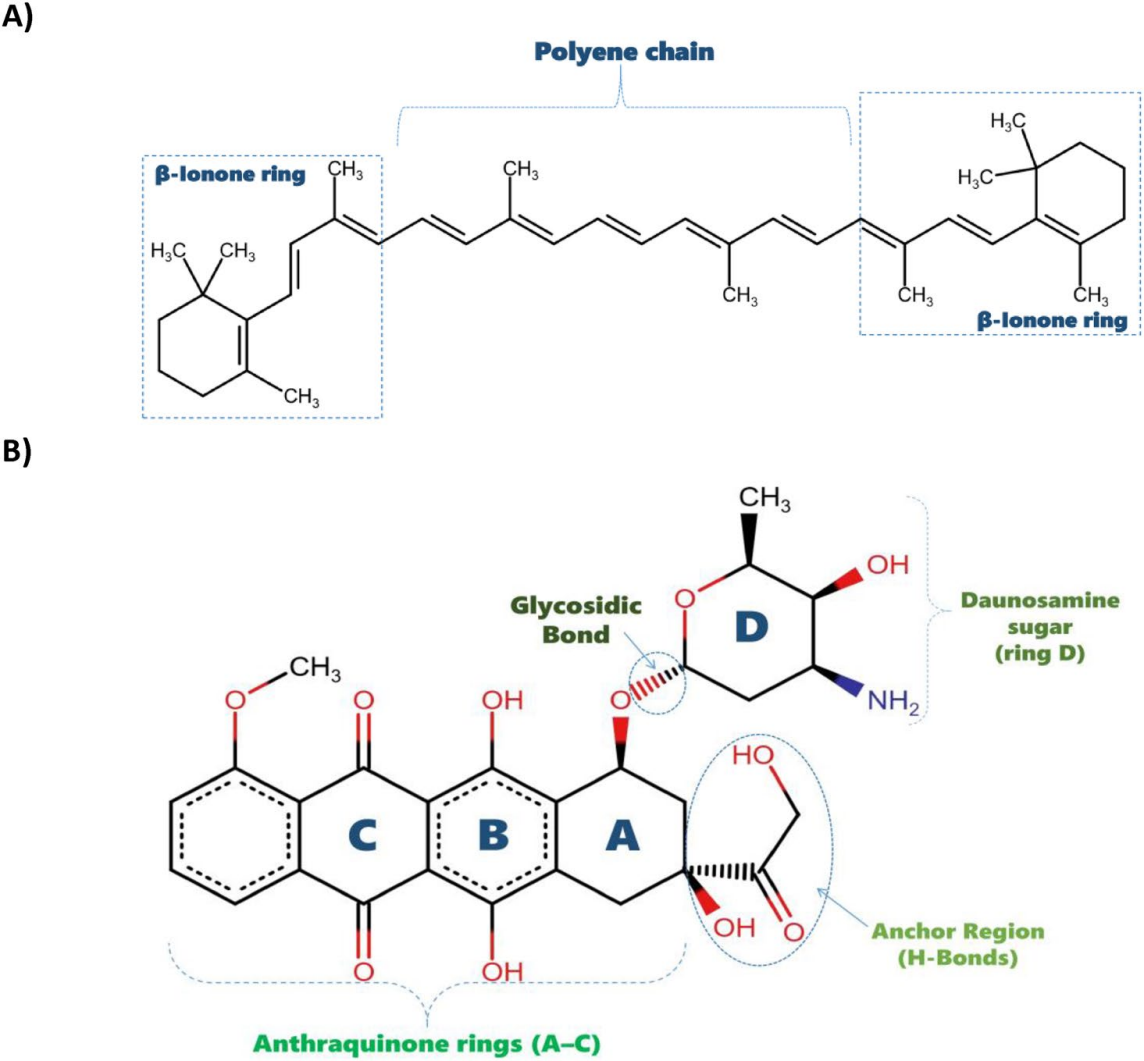
(**A**) Chemical structure of β-carotene, a symmetrical tetraterpene composed of a polyene chain with conjugated double bonds flanked by two β-ionone rings, enabling lipid membrane integration and antioxidant activity. (**B**) Chemical structure of doxorubicin, an anthracycline antibiotic featuring a tetracyclic quinone–hydroquinone chromophore linked to an amino sugar (daunosamine), which facilitates DNA intercalation and topoisomerase II inhibition.

Doxorubicin (Adriamycin®), an anthracycline antibiotic derived from *Streptomyces peucetius* var. *caesius*, is widely employed against solid and haematologic malignancies, including CRC and breast cancer^8^. Its structure is shown in (Fig. 1B). Doxorubicin acts by intercalating into DNA—preferentially at 5′-GC-3′ and 5′-CG-3′ sequences—impairing replication and transcription^9^. It also inhibits topoisomerase II by stabilising the DNA–enzyme complex, leading to double-strand breaks and apoptosis^9^. Additionally, its quinone/hydroquinone moieties undergo redox cycling to generate reactive oxygen species (ROS), enhancing cytotoxicity but contributing to cumulative cardiotoxicity^9^. While effective, doxorubicin’s side effects—myelosuppression, mucositis, and cardiac injury—limit its clinical utility and necessitate delivery strategies that preserve efficacy while minimising toxicity^1,9^.

Natural products provide a rich source of bioactive compounds, and carotenoids are potent antioxidants with chemopreventive potential^10,11^. Fig. 1A shows the structure of β-carotene, a lipophilic polyisoprenoid with a conjugated polyene chain and terminal β-ionone rings. It scavenges free radicals, quenches singlet oxygen, and is enzymatically converted into retinal, making it a key pro-vitamin A source^11–13^. Its structure allows incorporation into lipid bilayers, potentially stabilising membranes under oxidative stress^13^. Epidemiological studies associate higher β-carotene levels with reduced incidence of breast, colorectal, and other cancers^10,14^. Experimental studies corroborate their antiproliferative, pro-apoptotic, and anti-inflammatory effects^11,12,15,16^.

However, high-dose β-carotene supplementation has paradoxically increased lung-cancer risk in smokers and asbestos-exposed individuals, as demonstrated in the Alpha-Tocopherol, Beta-Carotene Cancer Prevention Study and the Physicians’ Health Study^17,18^. These findings stress the need for precise dosing and targeted delivery to balance therapeutic benefits and potential risks.

Despite extensive research on individual compounds, comparative evaluations of liposomal β-carotene and liposomal doxorubicin for CRC remain limited. Few studies have examined how each agent modulates liposomal bilayer properties, such as thermotropic behaviour, acyl-chain order, or head-group dynamics^6^. Moreover, cytotoxicity comparisons between these nano-formulations in CRC models such as HCT-116 cells are rare.

We introduce a neutral multilamellar liposomal system co-encapsulating β-carotene and doxorubicin (BC-DOX liposomes) and provide a comprehensive evaluation of its physicochemical properties, in-vitro anticancer efficacy against HCT-116 colorectal-carcinoma cells, and mechanistic insights via molecular docking and ADMET predictions. By directly comparing single-agent and co-delivery formulations, this work aims to (i) quantify the balance between cytotoxic potency and DNA-protective effects, (ii) characterise the influence of β-carotene on doxorubicin-mediated cell-cycle arrest and apoptosis, and (iii) validate key protein–ligand interactions that underpin observed biological outcomes. This integrated approach addresses critical knowledge gaps and lays the groundwork for safer, more effective nanotherapeutics in CRC treatment.

## Materials and methods

### Chemicals and reagents

**Fig. 2:**
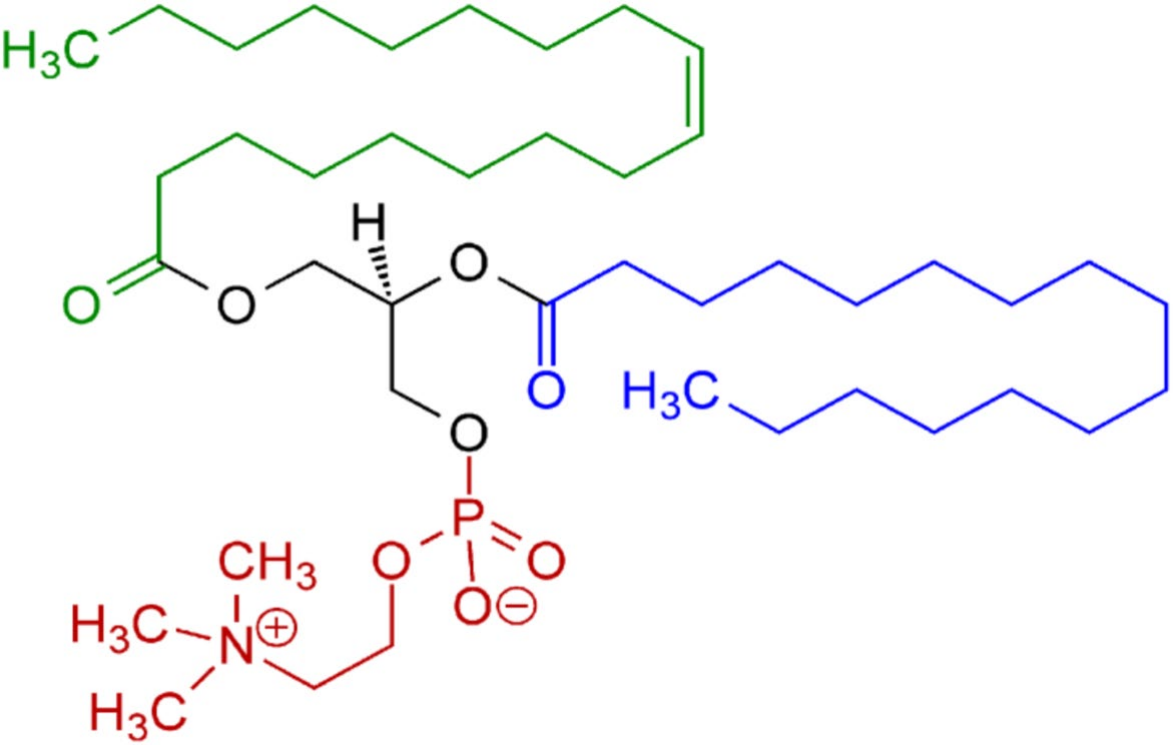
Schematic chemical structure of L-α-phosphatidylcholine (Soy Lecithin).

Β-carotene was extracted and purified from natural sources with spectral analysis confirming its molecular structure and molecular weight of 536.9 Da (Fig. 1A). Doxorubicin hydrochloride (molecular weight 543.52 Da) was purchased from Asta, Germany (Fig. 1B). Absolute ethanol (99.9% purity, HPLC grade) was obtained from DaeJung Chemicals, Korea. L-α-phosphatidylcholine (Soy Lecithin) powder was sourced from Carl Roth, Germany, with molecular weight 760 Da and ≥ 97% purity (Fig. 2). Phosphate-buffered saline (PBS, pH 7.4) was obtained from CDH, New Delhi.

HCT-116 human colorectal cancer cells were maintained in liquid nitrogen at -180 °C following American Type Culture Collection protocols and were obtained from Vacsera Vaccination Center, Cairo. DMSO (≥ 99.9% purity), DMEM medium, sodium bicarbonate, trypan blue (0.4%), penicillin/streptomycin (100 ×), trypsin–EDTA solution, glacial acetic acid, fetal bovine serum (FBS), sulphorhodamine-B (SRB), and trichloroacetic acid were procured from Sigma Chemical Co., USA. Additionally, 0.4% SRB solution in 1% acetic acid and isopropanol (100% purity) were prepared fresh before use. All aqueous solutions were prepared using distilled ultra-pure water (18.2 MΩ·cm) with HPLC-grade solvents and reagents stored according to manufacturer specifications.

### Liposome preparation and encapsulation efficiency

Neutral multilamellar vesicles (MLVs) were prepared using the thin film hydration method of Bangham^19^ with modifications for drug loading. The formulation comprised soy lecithin and doxorubicin at a 7:2 molar ratio (100 mg soy lecithin and 21 mg doxorubicin), with or without β-carotene at a 2:7 molar ratio relative to soy lecithin (20 mg β-carotene). All components were dissolved in 25 ml absolute ethanol in a 100 ml round-bottom flask and mixed thoroughly.

The organic solvent was removed by rotary evaporation under reduced pressure at 40 °C and 150 rpm until a uniform thin lipid film formed on the flask’s inner wall. The flask was further dried under vacuum for 2 h to remove residual solvent traces. The lipid film was hydrated with 10 ml phosphate-buffered saline (pH 7.4) pre-warmed to 37 °C, followed by gentle agitation in a 37 °C water bath for 15 min to promote film swelling. The flask was then mechanically shaken at 150 rpm for 60 min at 37 °C to form MLVs. After hydration, the flask was flushed with nitrogen gas and immediately sealed to prevent oxidation. Empty control liposomes were prepared identically using only soy lecithin.

Doxorubicin encapsulation efficiency was determined by separating unencapsulated drug through centrifugation at 6,000 rpm for 20 min at 4 °C. The supernatant containing free doxorubicin was collected, and drug concentration was measured spectrophotometrically at 485 nm using a UV–Vis spectrophotometer (Jasco V-630, Germany). Quantification was performed against a validated calibration curve (R^2^ ≥ 0.999) prepared in PBS.

β-carotene encapsulation efficiency was assessed following the method of Tan et al.^20^. One milliliter aliquots of β-carotene-loaded liposomes were vigorously vortexed with 3 ml ethyl acetate for 3 min at ambient temperature to extract free β-carotene. After centrifugation at 2,000 rpm for 5 min, the organic supernatant was collected. The extraction procedure was repeated twice to ensure complete recovery of unencapsulated β-carotene. The combined organic phases were diluted appropriately with ethyl acetate and quantified spectrophotometrically at 452 nm using ethyl acetate as empty. All measurements were performed in triplicate within the linear range of detection.

The entrapment efficiency (EE%) was calculated using the following equation^20^:

1 $$EE\%=\frac{Total drug imput\left({\text{mg}}\right)-Drug in supernatant({\text{mg}})}{Total drug input({\text{mg}})}\times 100$$

### Physicochemical characterization

#### Transmission electron microscopy

The morphology and size distribution of empty liposomes and drug-loaded formulations were analyzed using negative stain transmission electron microscopy (JEOL JEM-2100, Japan) operating at 200 kV. Samples were diluted 1:10 (v/v) in Tris buffer (pH 7.4) at 37 °C. Twenty microliters of diluted sample were applied to carbon-coated copper TEM grids (200 mesh) and allowed to adsorb for 1 min. Excess solution was removed by filtration, and grids were negatively stained with 1% phosphotungstic acid solution for 30 s. Images were captured at various magnifications to assess size distribution and structural integrity.

#### Dynamic light scattering and zeta potential analysis

Mean particle size, polydispersity index (PDI), and zeta potential of freshly prepared liposome formulations were determined using dynamic light scattering (Nanotrac Wave II, Microtrac, USA) at 25 °C. Samples were diluted in Tris buffer (pH 7.4) to achieve appropriate scattering intensity (50–200 kcps). Each measurement represented the average of three consecutive runs with automatic duration settings.

#### Differential scanning calorimetry

Thermal behavior of lyophilized liposome samples was investigated using differential scanning calorimetry (DSC-50, Shimadzu, Japan) calibrated with high-purity indium standard. Approximately 5 mg of lyophilized sample was sealed in aluminum pans with pierced lids and analyzed from 25 °C to 200 °C at a heating rate of 3 °C/min under nitrogen atmosphere (50 ml/min flow rate). An empty aluminum pan served as reference.

#### Fourier transform infrared spectroscopy

FTIR spectra were recorded using a Jasco FT/IR-4100 spectrometer (Tokyo, Japan) equipped with a DTGS detector. Lyophilized samples were mixed with spectroscopic grade potassium bromide (KBr) at a 1:100 ratio and pressed into transparent discs. Spectra were acquired at room temperature with 4 cm⁻^1^ resolution across the 4000–400 cm⁻^1^ range, averaging 64 scans per spectrum. Background spectra were collected using pure KBr discs. All physicochemical characterization experiments were conducted in triplicate, with data expressed as mean ± standard deviation.

### In vitro cellular and molecular effects of liposomal formulations

#### Cell culture and maintenance

HCT-116 colorectal cancer cells were cultured in RPMI-1640 medium supplemented with 10% fetal bovine serum, 100 U/ml penicillin, and 100 μg/ml streptomycin. Cells were maintained at 37 °C in a humidified atmosphere containing 5% CO₂. Cell viability was assessed using trypan blue exclusion assay, and only cultures with > 95% viability were used for experiments.

#### Cytotoxicity assessment by MTT assay

Cell viability was evaluated using the 3-(4,5-dimethylthiazol-2-yl)-2,5-diphenyltetrazolium bromide (MTT) colorimetric assay^21^. HCT-116 cells were seeded in 96-well plates at a density of 1 × 10^4^ cells/well in 100 μl complete medium and incubated for 24 h to allow cell attachment. After attachment, the medium was replaced with fresh medium containing test compounds at various concentrations prepared by two-fold serial dilution.

The cytotoxic potential of empty liposomes, free doxorubicin, free β-carotene, doxorubicin-loaded liposomes, β-carotene-loaded liposomes, and combination-loaded liposomes was evaluated in triplicate wells for each concentration. Cell monolayers were incubated for 48 h at 37 °C with 5% CO₂ under varying treatment concentrations.

Following treatment, drug-containing medium was aspirated, and cells were washed twice with sterile PBS. Fifty microliters of MTT solution (0.5 mg/ml in serum-free medium) was added to each well, followed by 4-h incubation at 37 °C. The MTT solution was then removed, and 100 μl of dimethyl sulfoxide (DMSO) was added to dissolve the formazan crystals. Plates were gently agitated for 10 min to ensure complete dissolution.

Absorbance was measured at 570 nm using a microplate reader (Boster Immunoleader, USA) with 630 nm as reference wavelength. Cell viability was calculated as percentage relative to untreated controls, and IC₅₀ values were derived from dose–response curves using four-parameter logistic regression analysis^22^.

Statistical analysis was performed using R software with Type III two-way ANOVA to assess the effects of formulation type, concentration, and their interaction on cell viability^23,24^. Post-hoc pairwise comparisons were conducted using estimated marginal means (EMMs) with Tukey’s adjustment for multiple comparisons, employing the emmeans package^25^. All experiments were performed in technical replicates.

#### Single-cell gel electrophoresis (comet assay)

DNA damage assessment was performed using the alkaline comet assay according to Singh et al.^26^ with modifications by Blasiak et al.^27^. This method detects various DNA damage types including single- and double-strand breaks, DNA adducts, cross-links, and alkaline-labile sites.

Cell microgels were prepared in three layers on pre-cleaned, charged microscope slides. The first layer consisted of 100 μl normal melting point agarose (0.7% in PBS) applied to slides and covered with coverslips. After solidification at 4 °C for 10 min, coverslips were removed. Low melting-point agarose (0.5% in PBS) was maintained at 37 °C and mixed with treated cell suspensions (approximately 1 × 10^4^ cells). One hundred microliters of this cell-agarose mixture was applied as the second layer, coverslipped, and solidified at 4 °C. A final protective layer of low melting-point agarose was applied, coverslipped, and allowed to solidify for 10 min at 4 °C before coverslip removal.

Slides were immediately immersed in freshly prepared lysis buffer (pH 10.0) containing 2.5 M NaCl, 100 mM EDTA, 10 mM Tris, 1% Triton X-100, 1% sodium hydroxide, and 10% DMSO, and incubated for 1 h at 4 °C in darkness. Following lysis, slides were drained and placed in DNA unwinding solution (300 mM NaOH, 1 mM EDTA, pH > 13) for 30 min at 4 °C to allow DNA denaturation.

Electrophoresis was performed in a horizontal gel electrophoresis chamber filled with fresh unwinding solution at 4 °C. Slides were subjected to electrophoresis at constant current (300 mA, approximately 1 V/cm) for 30 min. Post-electrophoresis, slides were neutralized with 0.4 M Trisma base (pH 7.5) for 10 min and stained with ethidium bromide (10 μg/ml).

Slides were examined using fluorescence microscopy (IX70; Olympus, Tokyo, Japan) with 549 nm excitation and 590 nm emission filters at 400 × magnification. Comets were scored visually, with each cell appearing as a nucleus (head) with a tail containing fragmented DNA. DNA damage was quantified by scoring 100 randomly selected cells per slide, and damage percentage was calculated based on the proportion of cells showing comet formation.

Statistical analysis was performed using triplicate experiments with data expressed as mean ± SD. One-way ANOVA was conducted to assess differences among treatment groups, followed by post-hoc comparisons using estimated marginal means (EMMs) with Tukey’s adjustment to control Type I error. Assumptions of normality and homoscedasticity were verified using standard diagnostic plots^24,28^. Statistical significance was set at p < 0.05.

#### Apoptosis detection by flow cytometry

Apoptotic cell death was quantified using Annexin V-FITC/propidium iodide (PI) double-staining followed by flow cytometric analysis. HCT-116 cells were seeded in 25 cm^2^ culture flasks at 1 × 10⁶ cells per flask and treated with IC₅₀ concentrations of all test formulations (empty liposomes, free doxorubicin, free β-carotene, doxorubicin-loaded liposomes, β-carotene-loaded liposomes, and combination-loaded liposomes) for 48 h.

Following treatment, cells were harvested by trypsinization, collected by centrifugation (2,000 rpm, 10 min), and washed twice with ice-cold PBS buffer. Cell pellets were resuspended in 100 μl Annexin V binding buffer containing 1 μl Annexin V-FITC and 5 μl propidium iodide staining solution, then incubated at room temperature in darkness for 15 min. Subsequently, 400 μl of binding buffer was added, and samples were analyzed immediately using a BD FACSCalibur™ flow cytometer.

Flow cytometric data were analyzed using appropriate gating strategies to distinguish viable cells (Annexin V⁻/PI⁻), early apoptotic cells (Annexin V⁺/PI⁻), late apoptotic cells (Annexin V⁺/PI⁺), and necrotic cells (Annexin V⁻/PI⁺). Results were expressed as apoptotic profiles showing the percentage of cells in each population.

Statistical analysis was performed using triplicate samples (n = 3) with Type III one-way ANOVA^23^ followed by estimated marginal means (EMMs) analysis with Tukey-adjusted pairwise comparisons^25^.

#### Cell cycle analysis

Cell cycle distribution was analyzed using propidium iodide staining and flow cytometry. HCT-116 cells were treated with IC₅₀ concentrations of all tested formulations for 48 h, then harvested and fixed in 70% ice-cold ethanol overnight at 4 °C. Fixed cells were washed twice with PBS and incubated with RNase A (1.5 μg/ml in PBS) for 1 h at 37 °C to remove RNA interference. Cells were then stained with propidium iodide solution (5 μg/ml) for 20 min on ice in darkness. Prior to analysis, cell suspensions were filtered through 40 μm nylon mesh to remove cell aggregates.

Flow cytometric analysis was performed using a BD FACSCalibur™ flow cytometer with appropriate gating to exclude debris and doublets. DNA content profiles were analyzed to determine the percentage of cells in G₀/G₁, S, and G₂/M phases of the cell cycle. Results were expressed as cell cycle distribution profiles.

Statistical analysis employed triplicate samples (n = 3) analyzed through Type III one-way ANOVA^23^. Post-hoc comparisons were conducted using estimated marginal means (EMMs) with 95% confidence intervals and Tukey’s adjustment to determine treatment effects on each cell cycle phase^25^.

### In silico study

#### Pharmacokinetic and toxicity profiles

The pharmacokinetic and toxicity profiles were predicted using complementary in-silico platforms to provide comprehensive ADMET assessment. Toxicity parameters including AMES mutagenesis, micronucleus formation, drug-induced liver injury (DILI), and carcinogenicity predictions were generated using ADMETlab 3.0, a web-based platform that integrates multiple machine learning models for toxicity prediction^29^. The remaining pharmacokinetic properties encompassing absorption (Caco-2 permeability, oral bioavailability, intestinal absorption), distribution (blood–brain barrier penetration, volume of distribution, plasma protein binding), metabolism (CYP interactions), and excretion (clearance, half-life) parameters were calculated using pkCSM, an established computational tool that employs graph-based signatures to predict small molecule ADMET properties^30^. This dual-platform approach leverages the specialized strengths of each tool to provide robust predictions across the complete spectrum of pharmacokinetic and safety parameters for comparative analysis of β-carotene (CID:5280489) and doxorubicin (CID:31703).

#### Structural identification and molecular docking

This study integrated experimental findings with computational approaches to elucidate the molecular mechanisms of action for doxorubicin (DOX) and β-carotene, focusing on validating their interactions with key molecular targets. Target identification was guided by prior experimental evidence, including cytotoxicity assays, apoptosis analysis, cell cycle profiling, and DNA damage assessments conducted on HCT-116 colorectal cancer cells. Based on these empirical findings, key cellular components were prioritized: the anti-apoptotic protein Bcl-2, Wnt signaling pathway protein β-catenin, drug efflux pump P-glycoprotein, and DNA replication enzyme Topoisomerase II. High-resolution three-dimensional structural templates were retrieved from the Protein Data Bank (PDB), specifically β-catenin (PDB ID: 1JDH), P-glycoprotein (PDB ID: 6QEX), and Topoisomerase II (PDB ID: 1ZXM), alongside relevant crystallographic structures for Bcl-2. InterProScan 5 was employed for genome-scale protein function classification and domain annotation^31^, enabling precise identification of conserved functional domains and critical binding sites, including Bcl-2 homology (BH) domains in anti-apoptotic proteins, Armadillo (ARM) repeats in β-catenin, and ATP-binding cassette (ABC) transporter domains in P-glycoprotein.

Chemical structures of investigative ligands were prepared using Open Babel toolbox^32^, facilitating geometry optimization and accurate molecular property representation, with key considerations including appropriate protonation states, correct tautomeric forms, and precise stereochemical configurations.

Molecular docking studies were performed using GNINA 1.3^33^, whose deep learning-based scoring functions incorporating convolutional neural networks offered enhanced accuracy in predicting binding poses and affinities, particularly relevant for conformationally flexible ligands like β-carotene. The protein–ligand interaction profiler (PLIP) 2021^34^ provided detailed binding mode characterizations through automated detection of non-covalent interactions, including hydrogen bonds, hydrophobic contacts, π-π stacking interactions, and salt bridges, while quantifying critical interaction geometries. BIOVIA Discovery Studio Visualizer^35^ generated high-quality 2D and 3D representations of protein–ligand complexes, complemented by PyMOL molecular graphics system^36^. Comparative binding affinity analysis evaluated distinct binding profiles across investigated protein targets, integrating structural data with energetic data to elucidate mechanistic insights regarding how different binding modes contribute to observed biological activities and therapeutic applications.

## Results and discussion

### Formulation and physicochemical characterization of drug-loaded liposomes

#### Entrapment efficiency and drug loading and morphological analysis (TEM)

**Fig. 3:**
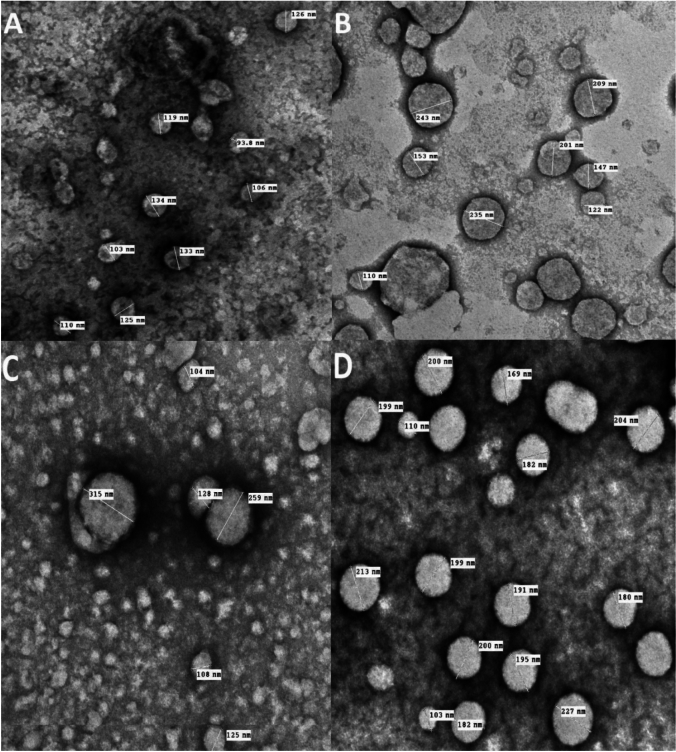
TEM images for empty liposomes (**A**), β-carotene-loaded liposomes (**B**), Doxorubicin-loaded liposomes (**C**) and Doxorubicin combined with β-carotene into liposomes (**D**).

The entrapment efficiency percentage exceeded 90% for all prepared liposomal suspensions when the drug was mixed with lipid powder before dissolving in ethanol. TEM images revealed that all liposomes exhibited almost spherical morphology with good dispersion and minimal aggregation for both empty and encapsulated β-carotene vesicles (Fig. 3).

Particle-size analysis by TEM showed that empty liposomes demonstrated a concentrated size distribution around 116.7 ± 14.08 nm, while β-carotene-doped liposomes ranged within 177.5 ± 51.14 nm (Fig. 3A,B). Doxorubicin-doped liposomes exhibited sizes of 182.8 ± 97.58 nm (Fig. 3C), with the increased particle size potentially attributed to electrostatic repulsive forces between lecithin PO₂⁻ groups and the hydroxyl (–OH) groups of doxorubicin. Liposomes co-encapsulated with β-carotene and doxorubicin measured 184 ± 34.3 nm (Fig. 3D).

TEM findings indicated that β-carotene or doxorubicin physically disrupted membrane-packing properties on liposomal surfaces (Fig. 3B–D), potentially inducing stronger lipid-bilayer interactions through hydrogen bonding. The presence of these compounds enlarged the spacing between neighboring bilayers, resulting in larger-sized liposomes compared to controls. This size increase may result from enhanced drug–lipid bilayer interactions via hydrogen bonding. These observations are explained by random distribution of non-polar β-carotene within the lipid bilayer without preferred orientation^37,80^, increasing the motional freedom of alkyl hydrocarbon chains and consequently expanding liposomal size. β-carotene insertion into the hydrophobic bilayer region supports these findings, demonstrating good agreement with DSC and FTIR results.

The measured particle size by TEM (184 nm) was notably larger than the DLS hydrodynamic diameter (122 nm), contrary to the common observation where DLS values exceed TEM due to hydration layers. This discrepancy may arise from multilamellar structures and artifacts during grid preparation, such as dehydration-induced fusion or vesicle collapse, which are known to inflate TEM diameters^38^.

#### Particle size distribution (DLS), polydispersity index (PDI) and zeta potential analysis

**Fig. 4:**
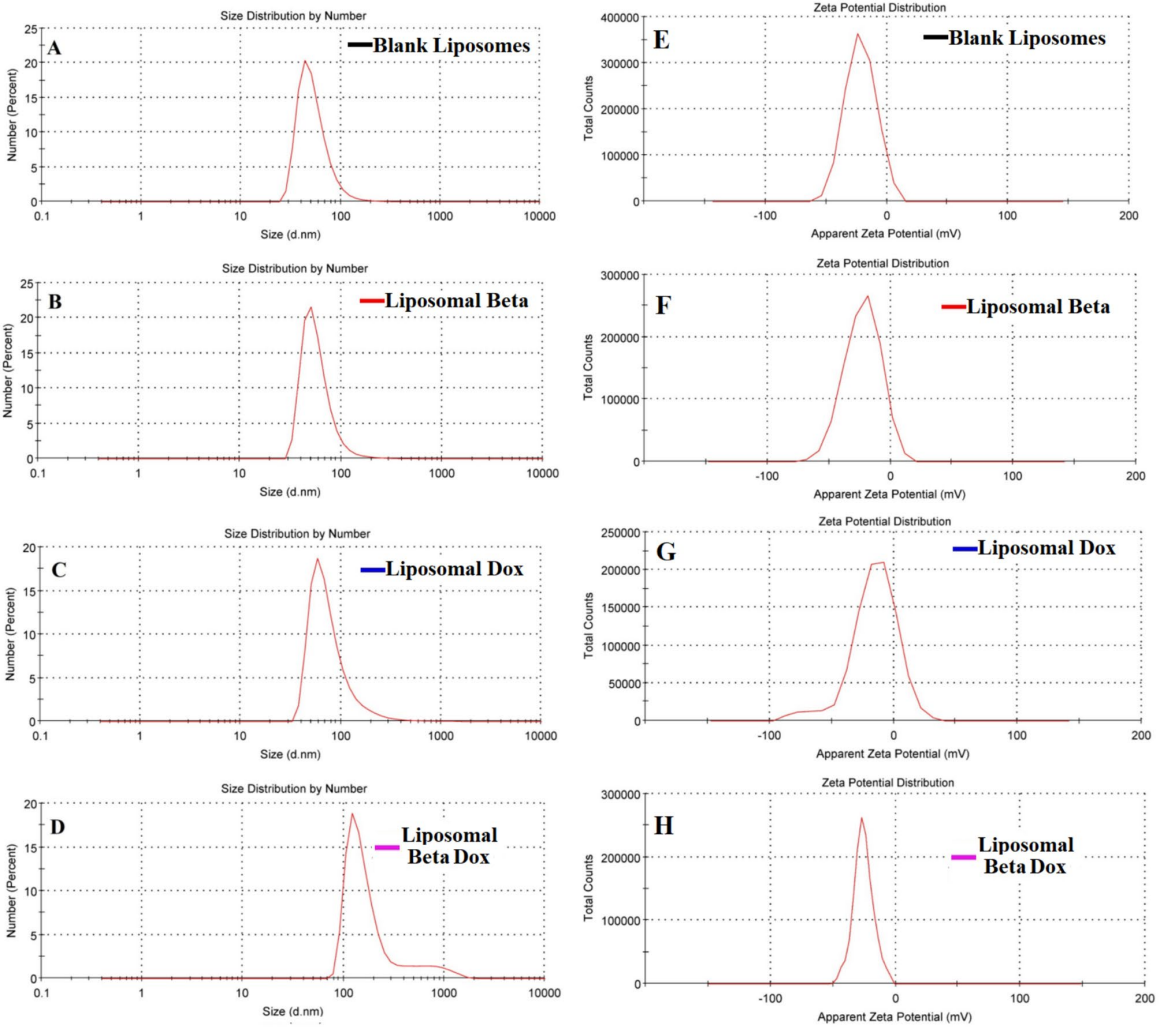
Liposomes size distribution measured by dynamic light scattering (DLS) for (**A**) empty soy lecithin liposomal sample, (**B**) β-carotene-encapsulated liposomes, (**C**) Doxorubicin-encapsulated liposomes and (**D**) Doxorubicin combined with β-carotene into liposomes. Zeta potential for (**E**) empty soy lecithin liposomal sample, (**F**) β-carotene-encapsulated liposomes, (**G**) Doxorubicin-encapsulated liposomes and (**F**) Doxorubicin combined with β-carotene into liposomes.

Dynamic light scattering (DLS) was employed to determine hydrodynamic diameter, while the polydispersity index (PDI) provided insight into colloidal uniformity (Table 1). PDI values above 0.7 typically indicate broad size distributions that compromise the accuracy and stability of DLS measurements^39^. As shown in Fig. 4A, pure soy-lecithin liposomes exhibited a mean hydrodynamic diameter of 43.82 ± 21.29 nm with a 0.411 PDI, suggesting narrow distribution and good homogeneity. Upon β-carotene incorporation, the mean vesicle diameter increased to 50.75 ± 28.54 nm with a 0.393 PDI (Fig. 4B). This slight increase indicates β-carotene molecules are intercalated within the lipid bilayer in a random orientation, enhancing bilayer fluidity and chain motional freedom.

**Table 1:** Summarized data obtained for the dynamic light scattering (DLS) and Zeta potential for liposomes before and after encapsulation by β-carotene, Doxorubicin, or Doxorubicin combined with β-carotene.

Sample name	Mean size diameter (nm) ± SD (nm)	PDI average	Mean zeta potential ± SD (mV)
Empty liposomes	43.82 ± 21.29	0.411	−21.5 ± 12.6
Liposomal β-carotene	50.75 ± 28.54	0.393	−22.6 ± 14.7
Liposomal Dox	78.82 ± 54.48	0.504	−15.8 ± 11.7
Liposomal beta Dox	122.4 ± 83.44	0.534	−25.6 ± 8.02

Doxorubicin encapsulation (Fig. 4C) produced a more pronounced shift, yielding a mean diameter of 78.82 ± 54.48 nm with 0.504 PDI. The size increase is attributed to electrostatic repulsion between the negatively charged phosphate (PO₂⁻) groups of lecithin and hydroxyl groups of doxorubicin, expanding inter-bilayer spacing. Co-encapsulation of doxorubicin and β-carotene at a 1:1 molar ratio (Fig. 4D) further increased the mean diameter to 122.4 ± 83.44 nm with a 0.534 PDI, indicating an additive effect of both molecules. β-carotene primarily influences bilayer expansion, while doxorubicin contributes to core hydration and overall vesicle enlargement, findings that align well with DSC and FTIR observations.

The PDI = 0.534 for the BC-DOX formulation approaches the upper limit for monodisperse systems but remains within the acceptable range for stable colloids. Although moderate polydispersity can influence reproducibility, it remains permissible when fabrication parameters are standardized^40,41^. The DLS results correlate well with TEM measurements (Fig. 3), confirming consistent particle-size trends between imaging and scattering techniques.

The magnitude of zeta potential is a key indicator of colloidal stability. Larger absolute values imply stronger electrostatic repulsion among particles, reducing aggregation and improving dispersion uniformity^42^. Consistent with previous studies, the empty soy-lecithin liposomes exhibited a moderately negative surface potential of –21.5 ± 12.6 mV (Fig. 4E)^43–46^, a characteristic value for phosphatidylcholine systems carrying residual phosphate groups.

Incorporation of β-carotene slightly enhanced the negative surface charge to –22.6 ± 14.7 mV (Fig. 4F), likely reflecting partial localization of the hydrophobic carotenoid near the lipid–water interface, which stabilizes the bilayer packing and strengthens surface charge density. Conversely, doxorubicin loading reduced the potential to –15.8 ± 11.7 mV (Fig. 4G). This attenuation is attributed to the cationic nature of doxorubicin, whose protonated amine groups interact with the negatively charged phosphate headgroups, partially neutralizing surface charges and lowering overall potential magnitude.

The co-encapsulated β-carotene–doxorubicin (BC–DOX) liposomes exhibited the most negative potential at –25.6 ± 8.02 mV (Fig. 4H). This enhanced charge separation suggests a synergistic structural arrangement in which β-carotene anchors within the hydrophobic core while doxorubicin associates with the polar headgroups, thereby restoring and amplifying electrostatic repulsion between vesicles. Such stabilization is consistent with the moderate PDI values obtained (≈ 0.53) and supports the formation of a relatively stable colloidal suspension.

Although a zeta potential of –25.6 mV indicates improved stability compared with single-drug formulations, it remains slightly below the ± 30 mV threshold typically regarded as optimal for long-term electrostatic stabilization. Nevertheless, systems within the –30 to + 30 mV range are often sufficiently stable under physiological ionic strength, particularly when additional steric factors (e.g., lipid packing or β-carotene reinforcement) contribute to interparticle repulsion^47^.

#### Thermal and molecular interaction analyses (DSC and FTIR)

Differential scanning calorimetry (DSC) and Fourier-transform infrared spectroscopy (FTIR) were employed to evaluate thermotropic behavior and molecular interactions of soy-lecithin liposomes in the presence of β-carotene and doxorubicin. These techniques reveal how encapsulated compounds modify bilayer order, phase transition temperature, and hydrogen-bonding networks within phospholipid assemblies^48–52^. Soy lecithin vesicles serve as model membranes closely resembling biological bilayers, and their thermal and vibrational responses provide valuable insight into formulation stability.

**Fig. 5:**
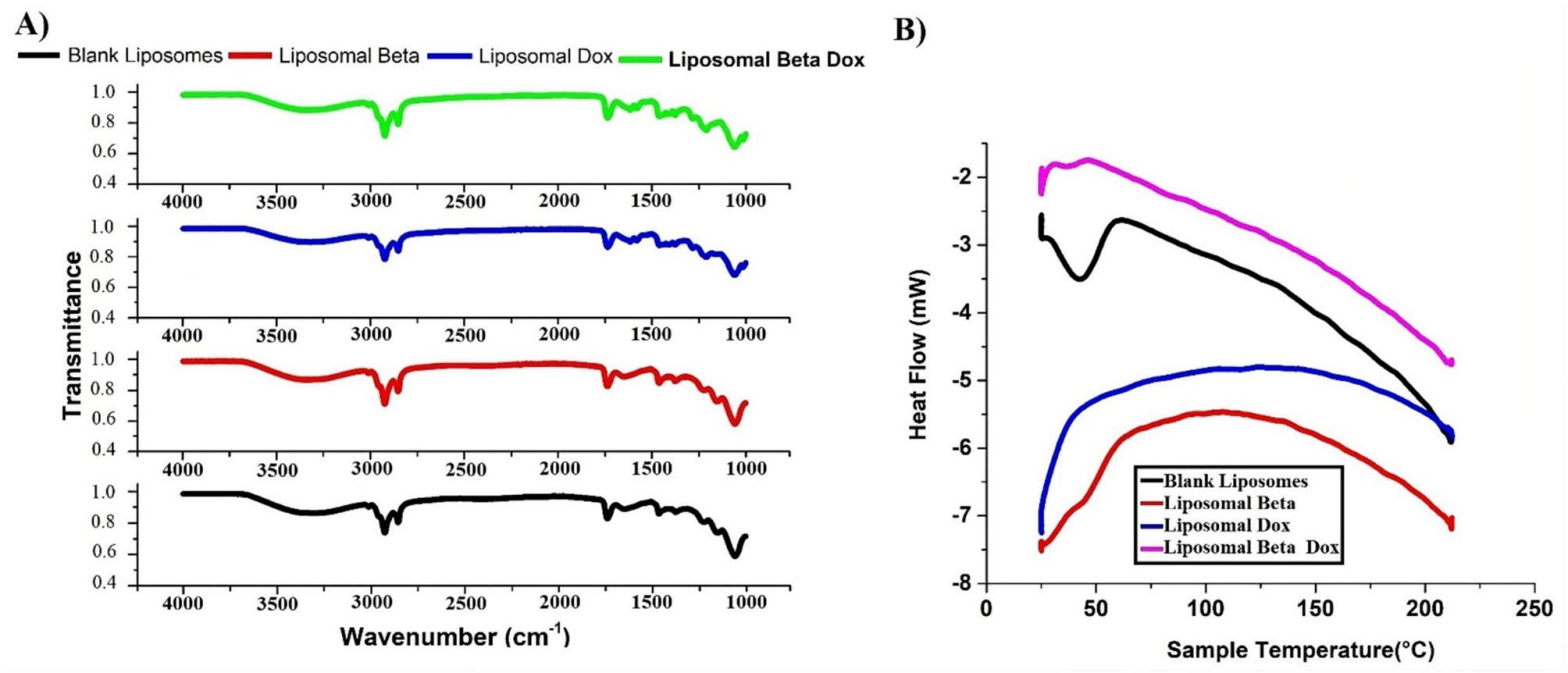
(**A**) DSC thermograms of pure soy-lecithin liposomes, β-carotene-loaded liposomes, doxorubicin-loaded liposomes, and β-carotene–doxorubicin co-loaded liposomes. (**B**) FTIR spectra of control and drug-loaded soy-lecithin liposomes in the 4000–400 cm⁻^1^ range.

Pure soy-lecithin vesicles showed a single cooperative endotherm at 43.76 °C, consistent with the gel-to-liquid-crystalline transition of phosphatidylcholine acyl chains (Fig. 5A) and in line with reported ranges for soybean PC systems^53–57^. Using this value as the reference temperature (T_m_,CTRL), incorporation of β-carotene shifted the peak upward to 44.77 °C. The absolute change was ΔT_m_ = + 1.01 °C, equivalent to a + 2.31% increase relative to control. At physiological temperature the membrane therefore remains well above its transition, with |T_m_ − 37 °C|= 7.77 °C for β-carotene liposomes compared with 6.76 °C for control. The direction and magnitude of this shift indicate tighter acyl-chain packing and slightly higher transition cooperativity, which is consistent with non-polar β-carotene intercalating into the hydrophobic core and restricting chain motion^58,59^.

Doxorubicin loading produced a qualitatively different thermal signature. The discrete endothermic peak at 43.76 °C became strongly attenuated and merged into baseline, preventing assignment of a sharp Tm. Peak collapse of this type reflects disruption of long-range acyl-chain cooperativity and generation of a broader distribution of local environments during heating. The observation is compatible with doxorubicin associating near the interfacial headgroup region through electrostatic and hydrogen-bonding interactions, which perturb ordered domains and blur the cooperative event rather than simply shifting it^51^.

Co-encapsulation of β-carotene with doxorubicin depressed the principal transition to 37.84 °C and broadened the profile. Relative to control the change was ΔT_m_ = − 5.92 °C, corresponding to a − 13.5% decrease. Notably, the distance from physiological temperature was |Tm − 37 °C|= 0.84 °C, placing the co-loaded membrane close to its cooperative region under biological conditions. A transition lying within one degree of 37 °C implies enhanced fluidity and defect density at assay temperature, which typically increases permeability and facilitates drug release, while the broadening indicates a reduction in cooperative unit size across the bilayer.

Structural modifications observed in DSC were further supported by FTIR spectroscopy, which probed vibrational changes in lipid functional groups. Spectra were recorded in the 4000–400 cm⁻^1^ region for pure and drug-loaded lyophilized soy-lecithin liposomes (Fig. 5B). The symmetric CH₂ stretching band of the acyl chains appeared at 2853 cm⁻^1^ in control liposomes and shifted slightly to 2852.6 or 2852 cm⁻^1^ upon doxorubicin or β-carotene incorporation, respectively, suggesting reduced gauche conformers and increased chain order^60^. In contrast, the co-loaded β-carotene–doxorubicin formulation exhibited a shift toward 2858 cm⁻^1^, consistent with increased membrane fluidity and partial destabilization in the gel phase.

The CH₂ antisymmetric stretching band moved from 2923 cm⁻^1^ (control) to 2920 cm⁻^1^ for β-carotene and β-carotene–doxorubicin samples, while the doxorubicin-only sample displayed 2926.6 cm⁻^1^, further indicating compositional effects on alkyl-chain dynamics^61,62^. The C = O stretching band near 1732 cm⁻^1^ shifted toward higher wavenumbers (1735–1741 cm⁻^1^) in drug-loaded samples, implying reduced hydrogen bonding at the ester carbonyl region due to drug insertion^60,63,64^. Similarly, the PO₂⁻ antisymmetric stretching band moved from 1241 cm⁻^1^ in control liposomes to 1228 cm⁻^1^ upon β-carotene incorporation, reflecting strengthened hydrogen bonding between polar headgroups and the carotenoid (Table 2).

**Table 2:** Summarizes the observed chemical shifts of major vibrational bands, confirming distinct structural rearrangements within the liposomal membranes.

Peak assignment	Wavenumber (cm^–1^)	Wavenumber (cm^–1^)			
		Control	Doxorubicin	Beta carotene	Dox mixed with beta
Symmetric stretching vibration of CH_2_ in acyl chain	(2800–2855)	2853	2852.61	2852	2858
Antisymmetric stretching vibration of CH_2_ in acyl chain	(2916–2921)	2923	2926. 61	2920	2920
Carbonyl stretching vibration C = O	(1730–1740)	1732	1735.58	1741	1735
Antisymmetric PO_2_^–^ stretching vibrations	(1215–1260)	1241	1241	1228	1241

These FTIR shifts collectively demonstrate that β-carotene primarily enhances acyl-chain ordering and stabilizes headgroup hydrogen bonding, whereas doxorubicin perturbs the interface by weakening hydrogen-bond networks and increasing fluidity. The combined system exhibits intermediate features, confirming mixed miscibility of both drugs within the bilayer matrix.

In the bilayer interior, the non-polar β-carotene molecules are embedded deep within the hydrophobic core, randomly distributed without fixed orientation, thereby enhancing motional freedom of lipid chains^37^. Conversely, doxorubicin localizes closer to the polar headgroup region, its cationic amines interacting electrostatically with phosphate moieties, leading to conformational disorder. These complementary positioning effects explain the DSC-observed reduction in Tm and the FTIR-detected increase in fluidity upon co-loading.

### In-vitro anticancer efficacy against HCT-116 colorectal cancer cells

#### Cytotoxicity (MTT assay)

The in vitro antiproliferative activity of the developed liposomal formulations was rigorously evaluated against HCT-116 colorectal carcinoma cells using an MTT assay over a 48-h incubation across a concentration range of 100–1200 µg mL⁻^1^. Tested groups included free β-carotene (BC), free doxorubicin (DOX), their respective liposomal encapsulations (BC-Lipo, DOX-Lipo), a dual-drug co-formulation (BC–DOX-Lipo), and an empty liposome vehicle control. Untreated cells were defined as 100% viable.

**Fig. 6:**
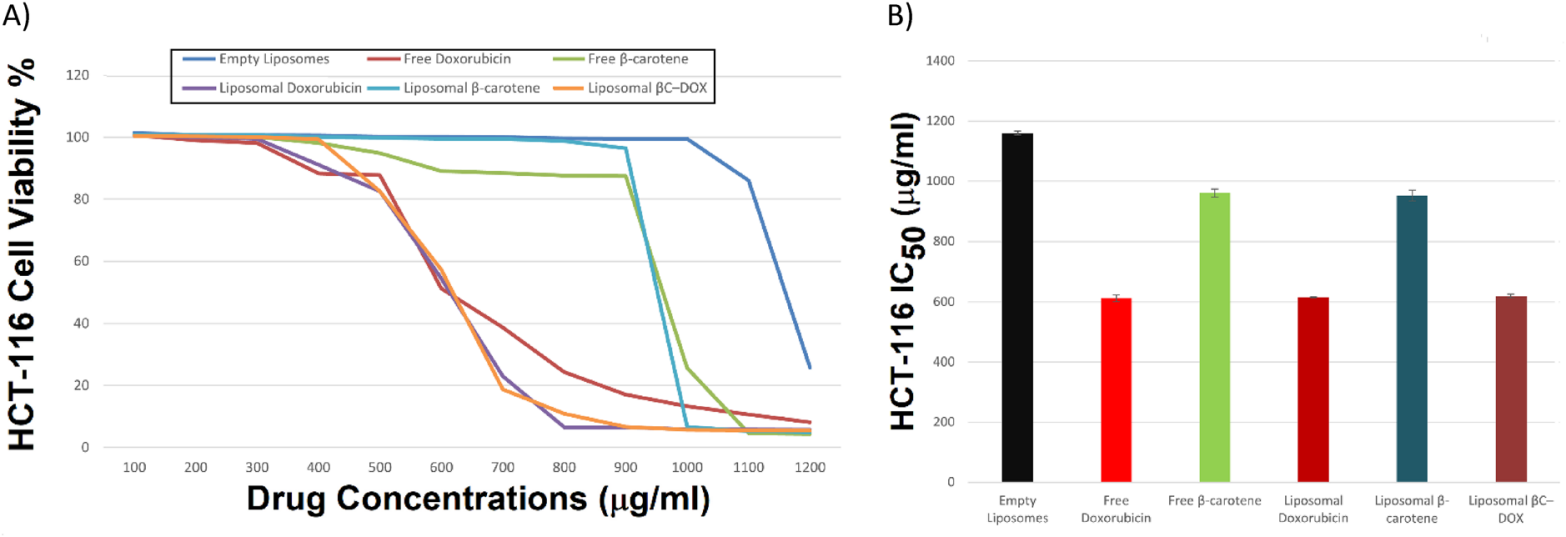
(**A**) In vitro cytotoxicity of empty liposomes, free doxorubicin, free β-carotene, and liposomes doped with either doxorubicin or β-carotene, or doxorubicin combined with β-carotene against colorectal carcinoma (HCT-116 cell line; incubated for 48 h with different drug concentrations starting from 100 to 1200 µg/mL). The MTT assay was used to measure cell viability. The results are the average ± standard error of three replicate studies. (**B**) IC₅₀ values for empty liposomes, free doxorubicin, free β-carotene, and liposomes doped with either doxorubicin or β-carotene, or doxorubicin combined with β-carotene against colorectal carcinoma (HCT-116 cell line by using MTT assay, 48 h post-treatment).

A two-way ANOVA (Fig. 6A) confirmed a highly significant main effect of concentration on cell viability (F (1, 60) = 187.05, p < 0.001) and a significant formulation–concentration interaction (F (5, 60) = 3.35, p = 0.0097), indicating non-parallel dose–response curves and justifying direct pairwise comparisons at each concentration.

Dose–response curves were steep for all doxorubicin-containing formulations and shallow for β-carotene–only and vehicle controls (Fig. 6A). At the maximum tested dose (1,200 µg mL⁻^1^), all drug-loaded formulations induced profound cytotoxicity, reducing cell viability to 5.07% (BC-Lipo), 5.76% (DOX-Lipo), and 5.49% (BC–DOX-Lipo). Free DOX produced comparable efficacy, lowering viability to 8.11%. In contrast, the empty liposome vehicle maintained 25.83% cell viability, demonstrating that the observed cytotoxicity is attributable primarily to the encapsulated agents rather than the lipid carrier.

At an intermediate dose (~ 600 µg mL⁻^1^), the release kinetics of encapsulated DOX became evident: liposomal DOX maintained 54.41% viability, slightly higher than free DOX (51.38%), consistent with controlled release from the vesicles. Both BC formulations exhibited markedly lower potency, sustaining viabilities of 89.01% (free BC) and 99.48% (BC-Lipo), respectively.

A Tukey HSD post hoc test confirmed significant differences between DOX-Lipo (5.87% viability) and empty liposomes (99.41% viability) at 1,000 µg mL⁻^1^ (mean difference = 93.54%, t = 6.37, P_adj_ < 0.001). Notably, the co-formulation (BC–DOX-Lipo) did not show significant superiority over DOX-Lipo alone (mean difference = –0.14%, P_adj_ > 0.99).

The definitive measure of cytotoxic potency, the half-maximal inhibitory concentration (IC₅₀), was derived from dose–response interpolation (Fig. 6B, Table 3). DOX-containing samples exhibited a tightly clustered IC₅₀ between 611 and 619 µg mL⁻^1^ (free DOX: 610.9 ± 11.4 µg mL⁻^1^; DOX-Lipo: 614.0 ± 1.8 µg mL⁻^1^; BC–DOX-Lipo: 618.9 ± 5.8 µg mL⁻^1^). In contrast, BC formulations required substantially higher concentrations to achieve comparable inhibition (free BC: 960.6 ± 13.6 µg mL⁻^1^; BC-Lipo: 951.8 ± 18.8 µg mL⁻^1^). The empty liposome control was essentially non-toxic, with an IC₅₀ of 1,159.8 ± 7.0 µg mL⁻^1^.

**Table 3:** Half-maximal inhibitory concentration (IC₅₀) data (Fig. 6B) establishing potency order for HCT-116 cells.

Formulation	IC₅₀ (µg mL⁻^1^) ± SD	Relative potency vs vehicle ( ×)
Empty liposomes	1,159.8 ± 7.0	–
Liposomal β-carotene	951.8 ± 18.8	1.22
Liposomal doxorubicin	614.0 ± 1.8	1.89
Liposomal β-carotene–doxorubicin	618.9 ± 5.8	1.87
Free β-carotene	960.6 ± 13.6	1.21
Free doxorubicin	610.9 ± 11.4	1.90

MTT reflects mitochondrial reductase activity and can diverge from apoptosis or cell-cycle readouts. Over 48 h, both free DOX and DOX-liposomes achieve comparable intracellular exposure, preserving IC₅₀, while the co-formulation deepens G₀/G₁ arrest and late apoptosis.

#### DNA damage assessment (comet assay)

**Fig. 7:**
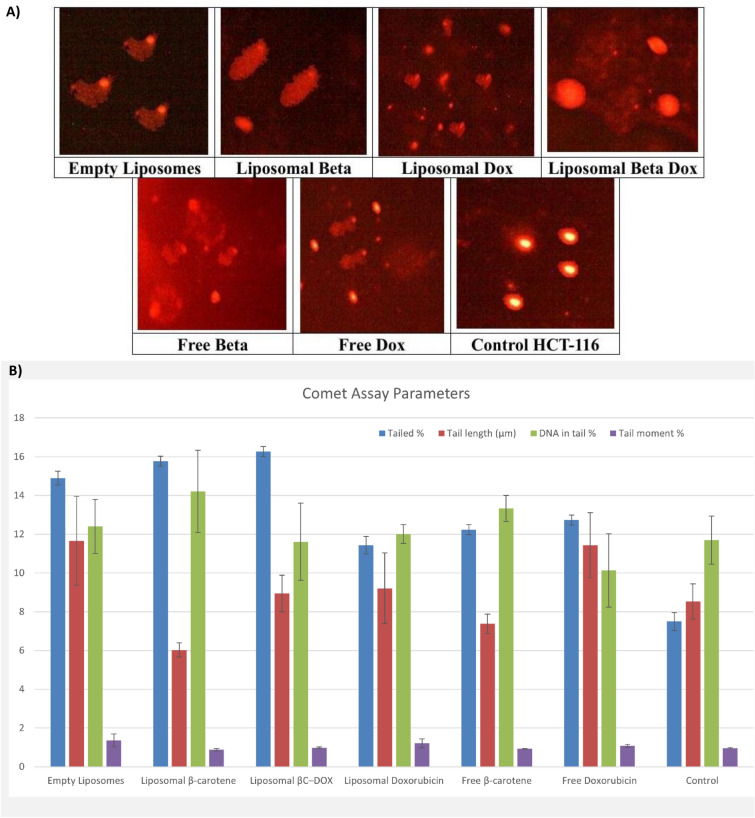
(**A**) Comet assay images of HCT-116 cell line (evaluation of DNA damage induced by empty liposomes, free Doxorubicin, free β-carotene, and liposomes doped with either Doxorubicin or beta carotene, or Doxorubicin combined with β-carotene compared to control HCT-116) (**B**) indicates the comet assay parameters (percentage tailed cells, tail length, percentage tailed DNA, and tail moment) for the control and post-treatment groups, and the differences between the control and the post-treatment groups.

Comet micrographs (Fig. 7A) revealed distinct DNA migration patterns among treatment groups. CTRL showed compact, round nucleoids with negligible tails, establishing baseline genomic integrity. EL produced short, fuzzy tails, reflecting mild vehicle-related stress. BC appeared diffuse with few discrete tails, suggesting limited uptake and weak genotoxicity. DOX generated bright heads with long, streaky tails, consistent with extensive DNA fragmentation. BC-Lipo yielded elongated, tadpole-like comets indicating moderate strand breaks, implying that liposomal encapsulation shifts β-carotene from antioxidant to mild pro-oxidant behavior in cancer cells. DOX-Lipo produced numerous small comets with visible tails, evidencing clear genotoxic insult but a more uniform pattern than free drug. In contrast, BC–DOX-Lipo displayed mostly round heads with minimal trailing, visually suggesting that co-encapsulated β-carotene attenuates DOX-associated DNA breaks.

One-way ANOVA revealed a highly significant treatment effect on tailed-cell % (F(6,14) = 242.93, p = 2.50 × 10⁻^13^) and tail length (F(6,14) = 6.42, p = 0.002) and a marginal effect on tail moment (F(6,14) = 3.74, p = 0.020). DNA in tail % did not differ significantly (F(6,14) = 2.22, p = 0.103), indicating that formulations modulated the frequency and spatial extent of migration rather than the proportion of fragmented DNA per nucleus—a kinetic rather than purely dose-dependent effect.

In Fig. 7B, Relative to CTRL (7.50 ± 0.46% tailed cells), EL increased damage to 14.90 ± 0.36% (p < 0.001), a 99% rise attributable to vehicle-induced oxidative stress. BC-Lipo showed 15.77 ± 0.25% (p < 0.001 vs CTRL), a 29% increase over free BC (12.23 ± 0.25%). DOX induced 12.73 ± 0.25% damage, while DOX-Lipo exhibited the highest frequency (16.27 ± 0.25%), a 117% rise vs CTRL and 27.8% over free DOX (both p < 0.001), reflecting enhanced uptake and prolonged nuclear exposure. BC–DOX-Lipo reduced tailed-cell % to 11.43 ± 0.45%, 30% lower than DOX-Lipo (p < 0.001), confirming that β-carotene mitigates DOX-induced oxidative DNA damage.

For tail length (µm), CTRL measured 8.53 ± 0.91. EL (11.66 ± 2.29) and DOX (11.43 ± 1.68) both exceeded CTRL (p < 0.05). DOX-Lipo (8.94 ± 0.95) did not differ from CTRL despite its higher tailed %, suggesting uniform, moderate fragmentation due to controlled release. BC-Lipo (6.02 ± 0.37) and BC (7.38 ± 0.50) produced shorter tails, while BC–DOX-Lipo (9.21 ± 1.82) remained near baseline, indicating attenuated fragmentation.

DNA in tail % values clustered without significance: CTRL 11.70 ± 1.24%, EL 12.40 ± 1.39%, BC-Lipo 14.21 ± 2.13%, DOX-Lipo 11.61 ± 2.00%, BC–DOX-Lipo 12.01 ± 0.48%, BC 13.33 ± 0.67%, DOX 10.13 ± 1.89%. Tail moment peaked for EL (1.366 ± 0.328; p < 0.05 vs most groups), exceeding CTRL (0.960 ± 0.029), BC-Lipo (0.874 ± 0.065), DOX-Lipo (0.972 ± 0.052), BC (0.929 ± 0.016), and DOX (1.077 ± 0.074). BC–DOX-Lipo recorded 1.210 ± 0.230, intermediate between control and high-damage treatments.

The invariance of DNA in tail % across all groups indicates that formulations influence intracellular delivery kinetics and damage frequency rather than the absolute burden of fragmented DNA per nucleus. Thus, uptake route, release dynamics, and subcellular localization—not total dose—drive the observed differences. EL’s elevated tail moment, despite lacking active drug, demonstrates that lipid vehicles contribute directly to genotoxicity via endocytosis-mediated membrane disruption and transient ROS generation.

The contrast between free BC and BC-Lipo reinforces this: free BC’s limited activity (12.23 ± 0.25%) stems from poor penetration, whereas BC-Lipo’s higher damage frequency (15.77 ± 0.25%) results from efficient intracellular delivery and conversion to a pro-oxidant within lipid-rich compartments where oxygen tension and metal availability favor oxidative reactivity.

DOX, entering by passive diffusion, rapidly intercalates DNA and poisons topoisomerase II, causing long tails in relatively few cells that experience acute nuclear exposure. Conversely, DOX-Lipo, internalized via endocytosis, releases its payload gradually post-endosomal escape, producing the highest tailed % (16.27%) but near-CTRL tail lengths (8.94 ± 0.95 µm)—a “many-cells/short-tails” profile of distributed, sub-maximal fragmentation.

BC–DOX-Lipo integrates these mechanisms through spatially segregated intracellular behavior. Co-encapsulation ensures co-uptake: β-carotene partitions into membranes and endomembrane interfaces where it quenches ROS, while doxorubicin diffuses to the nucleus to inhibit topoisomerase II. The resulting comet profile—11.43 ± 0.45% tailed cells (30% lower than DOX-Lipo), 9.21 ± 1.82 µm tail length (near CTRL 8.53 ± 0.91), and 1.210 ± 0.230 tail moment—indicates effective redox buffering that suppresses oxidative strand breaks without blocking DOX–DNA engagement.

#### Apoptosis induction and cell cycle modulation

To elucidate the mechanistic basis of cytotoxicity, we assessed programmed cell death and cell cycle perturbations in HCT-116 cells using Annexin V-FITC/PI staining and flow cytometry after 48-h treatment at IC₅₀ concentrations. For consistency with earlier sections, groups are abbreviated as: CTRL (untreated control), EL (empty liposomes), BC (free β-carotene), DOX (free doxorubicin), BC-Lipo (liposomal β-carotene), DOX-Lipo (liposomal doxorubicin), and BC–DOX-Lipo (co-encapsulated β-carotene and doxorubicin).

**Fig. 8:**
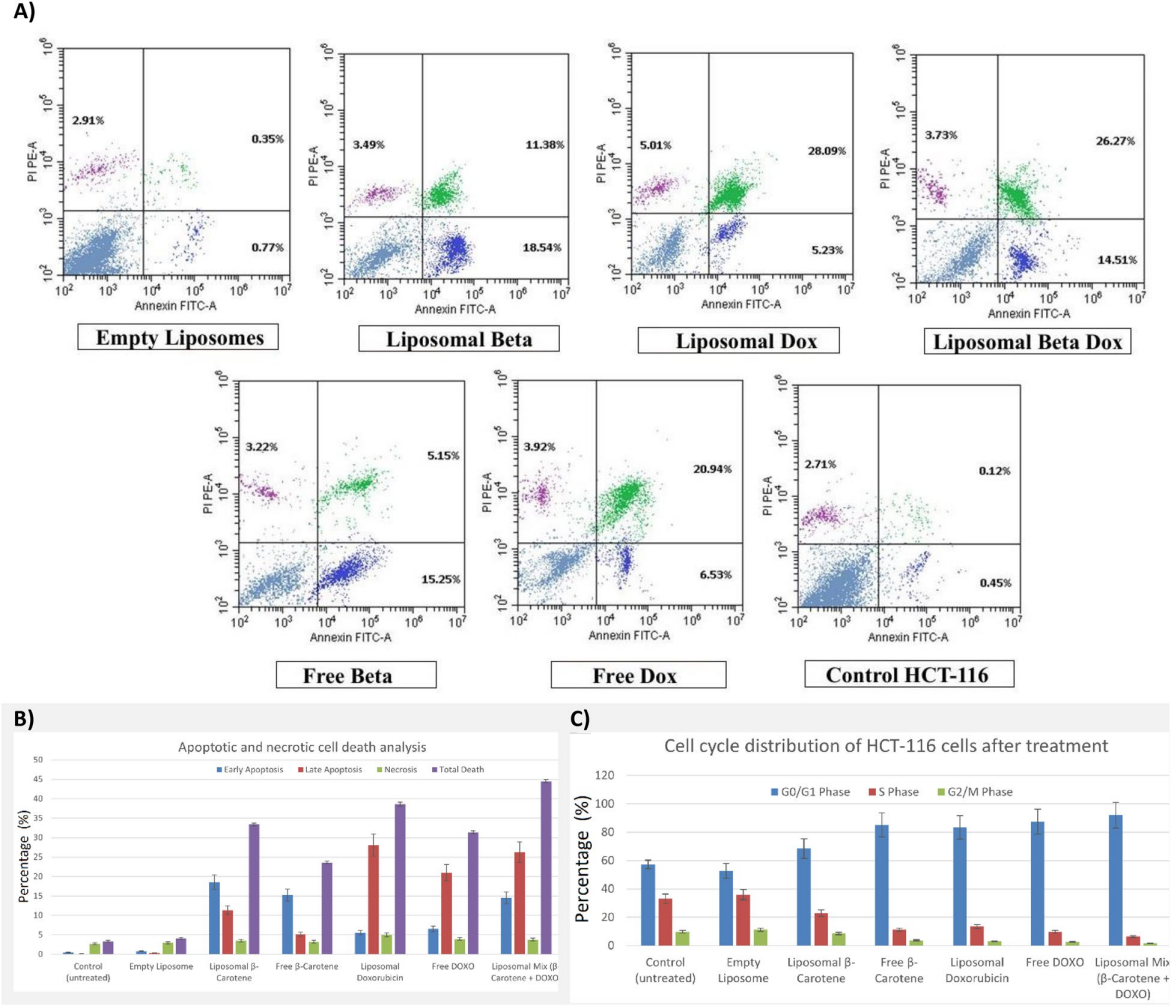
Flow cytometric analysis of apoptosis and cell cycle distribution in HCT-116 cells. (**a**) Representative flow cytometry plots of control HCT-116 cells and cells treated for 48 h with IC₅₀ concentrations of empty liposomes, free DOXO, free β-carotene, and liposomal formulations containing DOXO, β-carotene, or both. (**b**) Quantification of early apoptosis, late apoptosis, necrosis, and total cell death (mean ± SD, n = 3). Liposomal formulations significantly increased apoptotic fractions, with the Liposomal Mix (β-carotene + DOXO) showing the highest total death (44.51 ± 4.45%), indicating a synergistic pro-apoptotic effect (*p* < 0.05). (**c**) Cell cycle analysis showing G0/G1 arrest (92.06 ± 9.2%) induced by the Liposomal Mix, accompanied by reduced S and G2/M populations, suggesting enhanced cell-cycle blockade versus free drugs.

Flow cytometric analysis (Fig. 8A) revealed distinct apoptotic profiles across formulations. CTRL (0.45 ± 0.05% early apoptosis, 0.12 ± 0.02% late apoptosis; total death 3.28 ± 0.33%) and EL (0.77 ± 0.08% early, 0.35 ± 0.05% late; total death 4.03 ± 0.40%) showed negligible apoptosis, confirming that observed cytotoxicity is drug-induced and that the lipid vehicle exhibits minimal inherent toxicity. Necrosis remained consistently low across all groups (2.71–5.01%), indicating a targeted, programmed cell death mechanism rather than non-specific cytotoxicity.

In Fig. 8B, Liposomal formulations significantly enhanced pro-apoptotic activity relative to free drugs. DOX-Lipo induced total apoptosis of 38.62 ± 3.86% (5.52 ± 0.55% early, 28.09 ± 2.81% late), a statistically significant increase over free DOX (31.39 ± 3.14%; 6.53 ± 0.65% early, 20.94 ± 2.09% late; p < 0.05). The pronounced late apoptosis in DOX-Lipo (28.09%) versus DOX (20.94%) reflects sustained nuclear engagement and orderly execution of programmed death pathways characteristic of controlled intracellular release. Similarly, BC-Lipo (33.41 ± 3.34% total; 18.54 ± 1.85% early, 11.38 ± 1.14% late) was significantly more effective than BC (23.62 ± 2.36% total; 15.25 ± 1.53% early, 5.15 ± 0.52% late; p < 0.01), confirming that encapsulation enhances cellular uptake and converts β-carotene’s activity profile within the cancer cell microenvironment^65^.

BC–DOX-Lipo yielded the highest total apoptosis at 44.51 ± 4.45% (14.51 ± 1.45% early, 26.27 ± 2.63% late), significantly greater than all free formulations and BC-Lipo alone (p = 0.0011). Notably, the difference between BC–DOX-Lipo and DOX-Lipo was not statistically significant (p = 0.9305), indicating that at their respective IC₅₀ concentrations, both formulations achieve similarly potent apoptotic responses. However, the co-formulation’s elevated early apoptosis (14.51% vs. 5.52% for DOX-Lipo) alongside sustained late apoptosis (26.27%) suggests a broader engagement of apoptotic pathways and potentially earlier commitment to cell death.

Cell cycle analysis (Fig. 8C) revealed that cytotoxic effects are mechanistically linked to profound G₀/G₁ checkpoint arrest. CTRL distributed normally across phases (57.19 ± 3.0% G₀/G₁, 33.04 ± 3.3% S, 9.77 ± 1.0% G₂/M), while EL showed minimal perturbation (52.79 ± 5.3% G₀/G₁, 35.90 ± 3.6% S, 11.31 ± 1.1% G₂/M). All active drug formulations induced statistically significant G₀/G₁ accumulation (p < 0.01 vs. CTRL), confirming that encapsulation enhances cellular uptake and converts β-carotene’s activity profile within the cancer cell microenvironment, as previously demonstrated in breast cancer models^66^. BC induced 85.09 ± 8.5% G₀/G₁ arrest with correspondingly depleted S (11.27 ± 1.1%) and G₂/M (3.64 ± 0.4%) phases. DOX accumulated 87.55 ± 8.8% in G₀/G₁, reducing S to 9.78 ± 1.0% and G₂/M to 2.67 ± 0.3%. Liposomal formulations maintained or enhanced these effects: BC-Lipo arrested 68.52 ± 6.9% in G₀/G₁ (22.92 ± 2.3% S, 8.56 ± 0.9% G₂/M), while DOX-Lipo reached 83.37 ± 8.3% G₀/G₁ (13.46 ± 1.3% S, 3.17 ± 0.3% G₂/M).

BC–DOX-Lipo produced the most dramatic cell cycle blockade, accumulating 92.06 ± 9.2% of cells in G₀/G₁. While G₀/G₁ differences among the most effective treatments were not statistically significant from each other, a critical distinction emerged in the S phase. BC–DOX-Lipo reduced S phase to 6.37 ± 0.6%—significantly lower than DOX-Lipo (13.46 ± 1.3%; p = 0.0186)—and depleted G₂/M to 1.57 ± 0.2%. This exceptionally stringent G₁/S checkpoint blockade, unmatched by either agent alone or in free form, provides strong evidence for synergistic interaction. The co-formulation creates a more robust and impenetrable barrier to DNA synthesis entry, effectively trapping cancer cells in a state where repair is futile and progression is blocked, ultimately committing them to programmed cell death.

These data collectively demonstrate that: (i) doxorubicin is the principal cytotoxic driver, (ii) liposomal encapsulation enhances both DOX and BC bioactivity through improved uptake and controlled release, (iii) co-encapsulation preserves DOX potency (equivalent MTT IC₅₀) while amplifying downstream mechanistic endpoints, and (iv) the superior anticancer efficacy of BC–DOX-Lipo arises from synergistic reinforcement of the G₁/S checkpoint coupled with sustained apoptotic commitment. Mechanistically, this explains the disconnect between comet assay readouts and biological outcomes: BC–DOX-Lipo reduces acute DNA damage frequency (lower tailed % than DOX-Lipo) yet strengthens cell death pathways. The co-formulation redirects the cellular response from “violent breaks” toward “orderly exit”—β-carotene’s redox buffering tempers oxidative DNA damage while doxorubicin maintains nuclear engagement, shifting the phenotype from acute fragmentation to checkpoint-enforced programmed death. This manifests as deeper G₀/G₁ arrest, more stringent S phase depletion, and increased late apoptosis, representing redox-coupled modulation of genotoxic stress rather than simple damage amplification.

### In silico study

#### Comparative in-silico pharmacokinetic (ADMET/PK) and safety profiles

This analysis contextualizes the physicochemical differences between β-carotene (BC) and doxorubicin (DOX) within the formulation lipid phosphatidylcholine (PC) using computational ADMET predictions. We emphasize that these are in silico estimates intended to guide formulation design and interpretation, rather than in vivo pharmacokinetic measurements. The analysis complements the scope of our in vitro findings^67–78^.

β-Carotene exhibits substantially better predicted intestinal permeability (Caco-2 logPapp –4.63, Table 4) than doxorubicin (–6.93, Table 4) and is classified as orally bioavailable at both 20% and 50% thresholds (Table 4). This aligns with experimental human absorption studies demonstrating efficient uptake of provitamin A carotenoids when delivered in lipid-rich formulations^67^. By contrast, doxorubicin shows negligible oral uptake ("Non-Bioavailable", Table 4) and poor intestinal absorption ("Non-Absorbed", Table 4), consistent with its clinical use exclusively via intravenous routes^68^. The efflux liability for both compounds—each being a P-glycoprotein substrate (Table 4)—suggests that co-administration with efflux inhibitors might enhance systemic exposure, an approach under active investigation for overcoming chemotherapy resistance^69^.

**Table 4:** Comparative pharmacokinetic (PK) profile of β-carotene and doxorubicin.

Category	Property	Predicted (β-Carotene)	Predicted (Doxorubicin)	Unit/note
Absorption	Caco-2 permeability	−4.63	-6.93	**logPapp**
	Human oral bioavailability (20%)	Bioavailable	Non-bioavailable	
	Human intestinal absorption	Absorbed	Non-absorbed	
	Human oral bioavailability (50%)	Bioavailable	Non-bioavailable	
	P-glycoprotein substrate	Substrate	Substrate	
	Skin permeability	−3.34	0.3	**log Kp**
Distribution	Blood–brain barrier (BBB) penetration	Penetrable	Non-penetrable	
	Fraction unbound (Human)	2.45	0.83	**%**
	Plasma protein binding	74.93	76.23	**%**
	Steady state volume of distribution (Vss)	8.62	3.44	**L/kg**
Metabolism	CYP3A4 substrate	Non-Substrate	Substrate	
	CYP2C9 inhibitor	Inhibitor	Non-inhibitor	
	Breast cancer resistance protein (BCRP) inhibitor	Inhibitor	Non-inhibitor	
	OATP3B3 inhibitor	Inhibitor	Non-inhibitor	
Excretion	Half-life of drug	> 3hs (implied by low Cl)	< 3hs	

β-Carotene’s high lipophilicity (logP ≈ 7.7) underlies its extensive predicted volume of distribution (Vss ≈ 8.62 L/kg, Table 4), far exceeding doxorubicin’s more modest Vss ≈ 1.44 L/kg (Table 4). This propensity for tissue sequestration is advantageous for sustained antioxidant delivery but raises potential for adipose accumulation and vitamin A–related toxicity at high doses^70,71^. Crucially, β-carotene is predicted to cross the blood–brain barrier (“Penetrable”, Table 4)—supported by molecular dynamics simulations of carotenoid–membrane interactions^72^—whereas doxorubicin’s polar structure precludes CNS penetration ("Non-Penetrable", Table 4). These differences suggest β-carotene’s suitability for neuroprotective applications, whereas doxorubicin’s distribution profile limits it to peripheral tumor sites. Plasma protein binding is comparable, with β-carotene at 74.93% and doxorubicin at 76.23% (Table 4).

Doxorubicin is metabolized as a CYP3A4 substrate (Table 4), introducing a risk of interactions with other CYP3A4 modulators^73^. In contrast, β-carotene is predicted not to be a CYP3A4 substrate but acts as a modest CYP2C9 inhibitor and may also influence BCRP and OATP1B1 transporters, being an inhibitor for all three (Table 4)^74^. Such inhibitory activities warrant caution when combining high-dose β-carotene supplements with medications cleared by these pathways.

Doxorubicin’s higher clearance underpins its relatively rapid systemic elimination and a predicted half-life of less than 3 h (Table 4)—a feature that helps mitigate chronic off-target toxicity but necessitates frequent dosing schedules^75^. In contrast, β-carotene’s low predicted clearance value supports prolonged plasma persistence and an implied half-life greater than 3 h (Table 4), potentially reducing dosing frequency in prophylactic regimens.

β-Carotene is predicted to be non-mutagenic in the Ames assay (“Safe”, Table 5) and negative for micronucleus formation (“Safe”, Table 5), reflecting its antioxidant mechanism rather than direct DNA reactivity. Doxorubicin, however, shows strong Ames positivity (“Toxic”, Table 5) and micronucleus induction (“Toxic”, Table 5), consistent with its DNA intercalation and topoisomerase II inhibition—mechanisms central to both its anticancer efficacy and its risk of secondary malignancies^39^. This is further supported by the user text indicating p53 activation for doxorubicin.

**Table 5:** Comparative safety profile of β-carotene and doxorubicin.

Category	Property	Predicted (β-Carotene)	Predicted (Doxorubicin)
Genotoxicity	AMES mutagenesis	Safe	Toxic
	Micronucleus	Safe	Toxic
	p53 pathway activation	Negative (implied)	Positive (implied)
Organ toxicity	Drug-induced liver injury (DILI)	Safe (DILI I: Safe)	Toxic (DILI II: Toxic)
	Hepatotoxicity score (Conceptual)	≈ 0.08 (very low)	≈ 0.96 (high)
	Respiratory toxicity	Potential risk (Implied)	Minimal risk (implied)
Carcinogenicity	Carcinogenesis	Toxic	Safe
Other	Maximum tolerated dose (LD50) log(mg/kg)	−3.26	0.33

The predicted drug-induced liver injury (DILI) for β-carotene is low (“Safe” for DILI I, Table 5), reflected in a conceptual DILI score of ≈ 0.08, while doxorubicin is predicted to be toxic (“Toxic” for DILI II, Table 5), with a conceptual score near unity (≈ 0.96), in agreement with clinical observations of anthracycline-induced hepatotoxicity^76^. Interestingly, our models flag a “Toxic” carcinogenicity prediction for β-carotene (Table 5), echoing the paradoxical findings of large supplementation trials in smokers^71^. This suggests that, despite its benign genotoxic profile, high-dose β-carotene may promote tumorigenesis via pro-oxidant effects under certain conditions^77^. Doxorubicin, conversely, is predicted as “Safe” regarding carcinogenesis in this specific model output (Table 5), which contrasts with its known genotoxic mechanism and risk of secondary malignancies.

Doxorubicin shows minimal skin sensitization risk, supporting its formulation in topical applications for cutaneous malignancies^78^. β-Carotene, however, is flagged for potential respiratory toxicity—likely reflecting pro-oxidant pulmonary effects when inhaled as fine particulates. The provided CSV did not contain specific parameters for skin sensitization or respiratory toxicity for direct comparison in (Table 5).

β-Carotene’s oral bioavailability, predicted BBB penetration, extended tissue retention, and generally favorable safety profile in terms of genotoxicity and hepatotoxicity (Tables 4 & 5) position it as a promising nutraceutical or adjunct in neurodegenerative and chronic oxidative-stress disorders. However, its paradoxical carcinogenicity signal (Table 4) and solubility challenges mandate careful dose optimization and delivery engineering (lipid-based or nanoemulsions). Doxorubicin remains unrivaled in acute cytotoxic oncology regimens, with its rapid clearance and utility in localized interventions despite its well-characterized cardiotoxic, genotoxic (Table 4), and hepatotoxic liabilities.

#### Comparative in-silico molecular docking analysis

Interaction with Bcl-2 (Anti-apoptotic protein) exhibits a canonical Bcl-twofold composed of four conserved Bcl-2 homology (BH) domains (BH3–BH4), which mediate its anti-apoptotic function. The N-terminal BH4 domain (residues 3–25) regulates interactions with pro-apoptotic partners, while the BH3 motif (residues 33–45) forms an amphipathic helix essential for heterodimerization. Additional BH3 (residues 75–93) and BH2 (residues 326–337) domains stabilize a hydrophobic groove critical for binding BH3-only proteins. Structural modeling (Gene3D G3DSA:3.30.437.30) reveals a compact helical bundle with solvent-accessible grooves enabling selective interactions. Functional annotations identify the protein as a member of the Bcl-2-like superfamily, involved in intrinsic apoptosis and mitochondrial membrane association. Conservation across metazoans highlights the evolutionary significance of these domains.

**Fig. 9:**
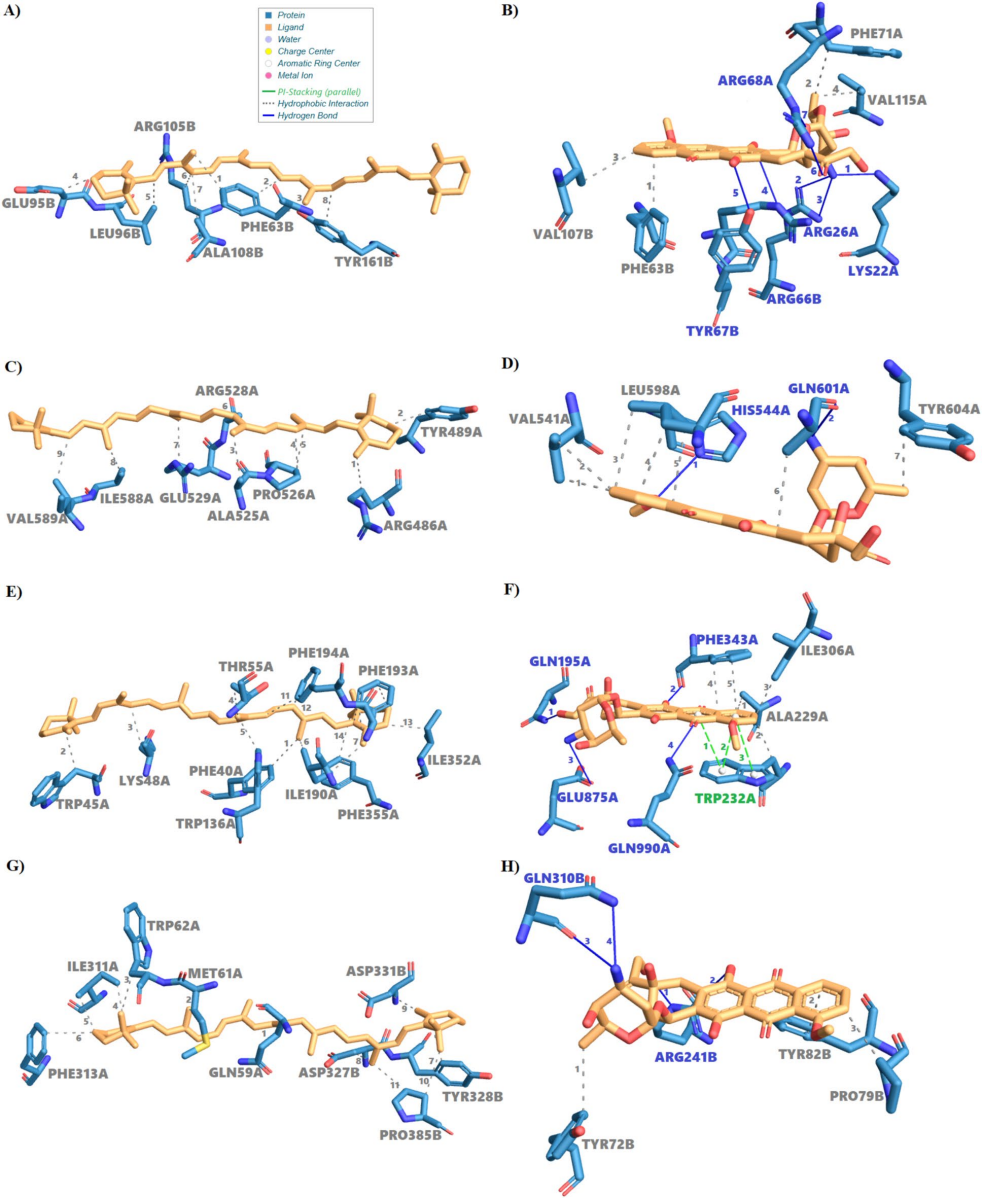
Comparative molecular docking analysis of β-carotene and doxorubicin with key cancer-related protein targets. (**A**) β-Carotene binds the BH3/BH2 hydrophobic groove of Bcl-2 via van der Waals interactions (Phe63, Leu96, Tyr363; 3.35–3.90 Å), mimicking BH3 peptides. (**B**) Doxorubicin spans chains A and B of Bcl-2, forming hydrogen bonds (Lys22, Arg26A, Arg66B, Tyr67B, Arg68A; 3.80–3.33 Å) and hydrophobic contacts (Val307B, Val335A, Phe73A), disrupting electrostatic interactions. (**C**) β-Carotene binds ARM 5–32 of β-catenin via van der Waals interactions (ARG486–VAL589), lacking hydrogen bonds, inducing allosteric modulation (ΔG = –5.96 ± 0.42 kcal/mol). (**D**) Doxorubicin targets ARM 7–8 of β-catenin, forming hydrophobic contacts and bidentate H-bonds (HIS544, GLN603), blocking TCF/LEF binding (ΔG = –7.37 ± 0.47 kcal/mol). (**E**) β-Carotene forms 34 van der Waals interactions with P-glycoprotein (Phe40–Val589; 3.24–3.97 Å), lacking hydrogen bonding, suggesting allosteric modulation. (**F**) Doxorubicin forms hydrophobic, hydrogen bond, and π–π stacking interactions with P-glycoprotein (Trp232, Gln395, Glu875), enabling strong binding and efflux activation (ΔG = –9.15 ± 0.93 kcal/mol vs. –7.82 ± 0.49 kcal/mol). (**G**) β-Carotene binds across chains A and B of Topoisomerase II via hydrophobic contacts (Trp62A, Phe333A, Tyr328B; 3.29–3.93 Å), lacking hydrogen bonds. (**H**) Doxorubicin forms hydrogen bonds with Arg243B and Gln330B of Topoisomerase II, hydrophobic interactions (Tyr72B, Pro79B, Tyr82B), and covalent bonds (Ser337, Leu335), stabilizing its confined, high affinity pose.

Figure 9A shows β-carotene engaging Bcl-2 exclusively through hydrophobic interactions within the BH3/BH2 groove. Eight van der Waals contacts are formed at 3.35–3.90 Å, notably with Phe63 (three contacts), Leu96 (3.35 Å), and Tyr363 (3.63 Å). The ligand nests into the groove, mimicking the binding mode of native BH3 peptides. This passive inhibition likely stabilizes Bcl-2's conformation by competitively displacing pro-apoptotic ligands without disrupting internal electrostatic networks. Such a mechanism aligns with epidemiological evidence linking carotenoids to reduced oxidative stress and apoptosis modulation^23^.

Figure 9B presents doxorubicin’s hybrid binding profile, spanning both chains A and B. It forms four hydrophobic contacts (e.g., Val335A at 3.62 Å) and seven hydrogen bonds, including Lys22, Arg26A, Arg66B, and Tyr67B, with bond lengths of 3.80–3.33 Å. These involve polar residues beyond the core groove and exhibit optimal donor angles (300.5°–372.8°), indicating strong electrostatic stabilization. Docking scores confirm higher affinity (–9.06 ± 0.55 kcal/mol) compared to β-carotene (–7.83 ± 0.29 kcal/mol). This multi-domain engagement likely induces allosteric conformational changes that disrupt native electrostatic networks, explaining doxorubicin’s pro-apoptotic efficacy and associated cardiotoxicity^79^.

Interaction with β-catenin (Wnt signaling pathway—PDB: 3JDH) reveals a multidomain architecture central to its signaling role. The 3JDH_3 chain (529 residues) adopts a canonical Armadillo (ARM)/β-catenin fold, composed of twelve tandem ARM repeats as identified by SMART (SM00385) and Pfam (PF00534). These repeats form a superhelical scaffold with a hydrophobic groove that accommodates protein and small-molecule ligands. Domain classification via Gene3D (G3DSA:3.25.30.30) and SUPERFAMILY (SSF48373) places this region in a β-catenin functional family (FunFam) crucial for mediating intracellular protein interactions. The smaller 3JDH_2 chain (38 residues) contains a CTNNB3-binding domain (Pfam PF08347), annotated by PANTHER (PTHR30373), and is involved in β-catenin’s nuclear translocation and interaction with TCF/LEF transcription factors. Collectively, these domains allow β-catenin to transduce Wnt signaling into transcriptional regulation.

Figure 9C shows β-carotene binding across ARM repeats 5–32 via nine van der Waals interactions (3.33–3.95 Å) with residues such as ARG486, TYR489, ALA525, PRO526, GLU529, ILE588, and VAL589. Its elongated polyene chain lacks hydrogen bonding capacity, contributing to a lower binding energy (ΔG = –5.96 ± 0.42 kcal/mol). Although weaker, this interaction may induce subtle allosteric shifts in β-catenin’s superhelical architecture, modulating its activity without full inhibition. The absence of polar anchoring suggests flexible ligand positioning, consistent with β-carotene’s reported role in fine-tuning Wnt signaling through dietary modulation.

Figure 9D illustrates doxorubicin occupying a specific hydrophobic subsite within ARM repeats 7–8. It forms seven hydrophobic contacts (e.g., VAL543, LEU598, GLN603, TYR604; 3.32–3.90 Å) and two strong hydrogen bonds—a bidentate interaction with HIS544 (2.05 Å, 373.5°) and GLN603 (2.38 Å, 359.3°). This tight polar anchoring stabilizes the complex (ΔG = –7.37 ± 0.47 kcal/mol), likely occluding the TCF/LEF binding interface and disrupting transcriptional co-activator recruitment.

Interaction with P-glycoprotein (PDB: 6QEX—Drug efflux pump) reveals functional specialization across multiple chains. Chain 6QEX_3 (residues 3–3,280) harbors an ATP-binding cassette (ABC) transmembrane domain (PF00664; E-value: 3.6 × 10⁻⁷^5^) and a P-loop NTP hydrolase domain (E-value: 5.3 × 10⁻^3^⁷), supporting ATP-dependent substrate transport (Reactome R-HSA-5630785; MetaCyc PWY-6403). Phobius predicts extracellular segments (residues 209–233, 854–858), suggesting roles in substrate recognition. Chains 6QEX_2 and 6QEX_3 (residues 3–225 and 3–220, respectively) contain immunoglobulin-like domains (PF07654/IPR003597; E-values ≤ 2.7 × 10⁻⁹), associated with cell adhesion and immune signaling (Reactome R-HSA-398933), indicating dual transport and immunomodulatory functionality.

Figure 9E depicts β-carotene’s binding to the hydrophobic core of P-glycoprotein. The ligand establishes 34 van der Waals interactions (3.24–3.97 Å) with residues such as Phe40, Trp45, Lys48, Ile390, and Phe355. No hydrogen bonds or π–π stacking interactions are observed. This broad, lipophilic contact pattern suggests a passive binding mechanism, potentially modulating transporter activity via membrane perturbation rather than competitive inhibition. The docking score (ΔG = –7.82 ± 0.49 kcal/mol) reflects this diffuse, lower-affinity engagement, aligning with previous reports of carotenoids indirectly influencing efflux kinetics.

Figure 9F illustrates doxorubicin’s high-affinity interaction (ΔG = –9.15 ± 0.93 kcal/mol). It binds within a defined subsite, forming five hydrophobic contacts (Ala229, Trp232, Ile306, Phe343), four hydrogen bonds (Gln395, Phe343, Glu875, Gln990), and three π–π stacking interactions with Trp232 (3.75–4.33 Å centroid distances, ~ 32° angles). These polar and π-stacking interactions stabilize doxorubicin’s planar anthracycline rings and are critical for high-affinity recognition. Anchoring to polar residues within the ABC domain supports active efflux by inducing conformational transitions.

Interaction with Topoisomerase II (PDB: 3ZXM—Doxorubicin’s primary target) with doxorubicin revealed a high-affinity binding interaction (ΔG = –9.30 ± 1.12 kcal/mol), driven by a combination of polar, hydrophobic, and covalent forces. As illustrated in Fig. 9H, doxorubicin binds within a well-defined pocket on chain B, where its planar anthracycline core forms hydrophobic interactions with Tyr72, Pro79, and Tyr82 (3.20–3.75 Å), stabilizing the aromatic scaffold. Crucially, the complex is anchored by four directional hydrogen bonds: two with Arg243 (2.58 Å, 2.66 Å) and two with Gln330 (2.74, 3.03 Å), enhancing specificity and binding strength.

In addition to these non-covalent interactions, doxorubicin exhibits covalent bonding with Ser337 and potential reactivity with Leu335, forming a geometrically constrained interaction mode. These covalent contacts may mimic the drug’s mechanism in stabilizing DNA-topoisomerase II cleavage complexes, thereby inhibiting DNA religation during chemotherapy. This dual anchoring strategy—hydrogen bonding and covalent linkage—effectively locks the ligand into a catalytically disruptive pose, targeting critical residues like Arg243 and Gln330.

In contrast, Fig. 9G shows β-carotene adopting a dispersed, hydrophobic binding mode with moderate affinity (ΔG = –7.62 ± 0.28 kcal/mol). Its extended polyene chain spans both chains A and B, forming eleven van der Waals contacts (3.29–3.93 Å) with residues including Trp62A, Phe333A, Tyr328B, and Pro385B. While lacking hydrogen bonds, the molecule engages in π-alkyl interactions and weak electrostatic contacts with Asp327 and Asp333, contributing modestly to stability.

These contrasting modes underscore 3ZXM’s capacity for ligand versatility. Doxorubicin’s localized, high-affinity binding highlights key pharmacophores for inhibitor design, while β-carotene’s broad, hydrophobic engagement supports strategies for developing allosteric modulators with reduced cytotoxic potential.

**Fig. 10:**
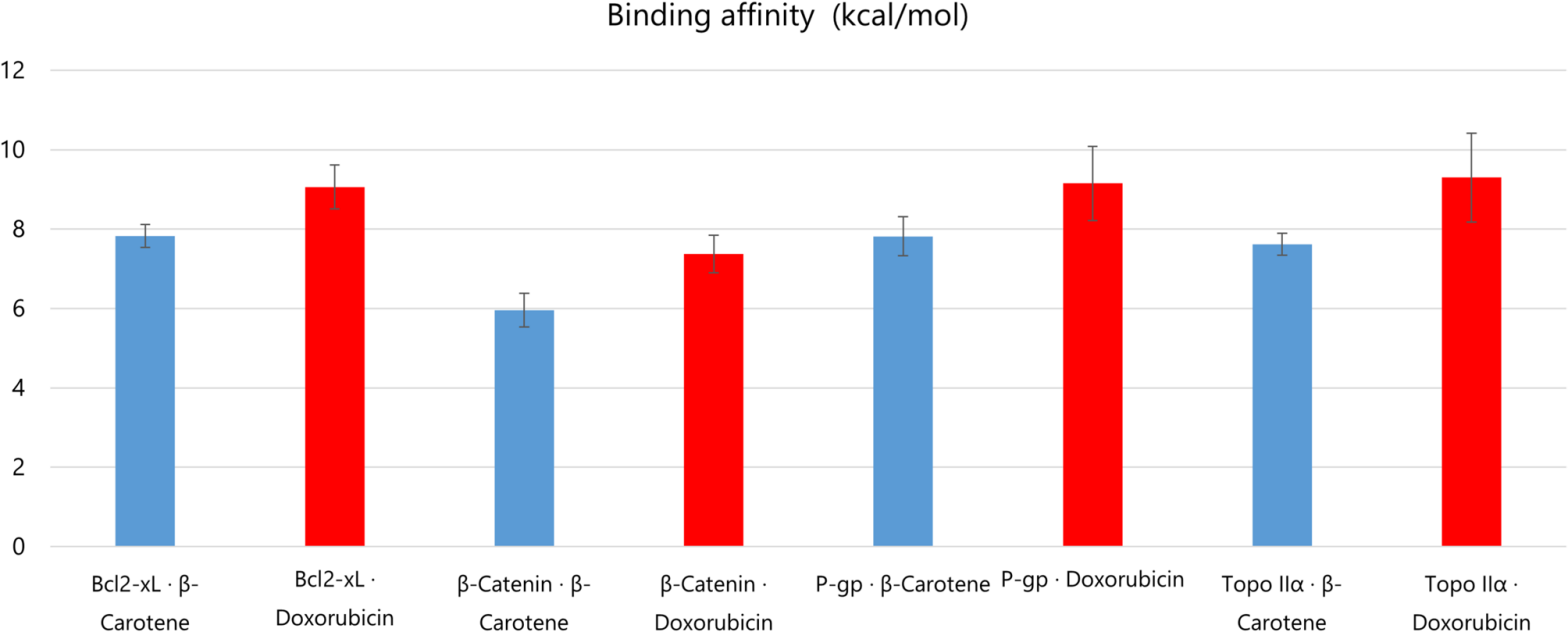
Comparative binding affinity analysis of DOX and β-carotene against four cancer-related protein targets. Bars represent mean binding affinities (|kcal/mol|) derived from the top 20 docking poses; error bars show SD (n = 20). Red bars: DOX; Blue bars: β-carotene. Higher bars indicate stronger interactions.

While doxorubicin (DOX) consistently demonstrates stronger binding affinities across all evaluated protein targets, β-carotene presents a compelling therapeutic profile characterized by selective interactions and significantly reduced toxicity. Docking analyses revealed that β-carotene engages key cancer-associated proteins with biologically meaningful affinities: –7.83 kcal/mol for Bcl2-xL, –7.82 kcal/mol for P-glycoprotein, and –7.62 kcal/mol for Topoisomerase IIα. Although lower than DOX (ranging from –9.06 to –9.30 kcal/mol), these values suggest that β-carotene can modulate target function without inducing the severe cytotoxicity associated with DOX’s high-affinity, multi-domain interactions. As illustrated in (Fig. 10), β-carotene exhibits a consistent binding profile across diverse targets, relying predominantly on extensive hydrophobic interactions and avoiding the polar and covalent anchoring seen with DOX. This less aggressive binding strategy may contribute to its safety and tolerability, reducing the risk of off-target disruptions. In contrast, DOX engages in hydrogen bonding and π–π stacking, often forming covalent-like contacts that explain its potent cytotoxicity and cardiotoxic effects. β-carotene’s favorable pharmacodynamic properties—membrane permeability, antioxidant capacity, and multi-target engagement—position it as a promising scaffold for structure-guided optimization. Chemical modifications could enhance its affinity while maintaining its safety advantages. Moreover, the narrow affinity range observed across targets implies a balanced mechanism of action, potentially minimizing systemic side effects common in traditional chemotherapeutics.

## Limitations and future perspectives

This study is limited to a single CRC line (HCT-116) without non-tumorigenic epithelial comparators or 3-D/orthotopic models. All efficacy data are in-vitro; the in-silico ADMET section provides computational context rather than in-vivo pharmacokinetics. The lecithin vehicle showed modest intrinsic bioactivity in comet readouts, underscoring the importance of optimized lipid composition and rigorous blank controls in follow-up work. Apparent “synergy” is better described as mechanistic enhancement: BC–DOX-Lipo preserved DOX IC₅₀ while deepening G₀/G₁ arrest and late apoptosis and moderating acute DNA fragmentation; a formal combination-index analysis has not yet been performed. The DSC down-shift and broadening in co-loaded liposomes suggest increased fluidity near 37 °C, which may favor release but raises stability questions. To address this, future work will (i) implement colloidal stability studies (size/PDI/ζ) at 4 °C and 25 °C, (ii) quantify serum and pH-dependent release at 37 °C (pH 7.4 and 6.5), and (iii) conduct ICH-aligned stability testing after finalizing the dosage form (suspension vs lyophilizate). Route of administration remains open; DOX is not orally bioavailable, whereas BC is. If oral colon targeting is pursued, gastric protection and colon-triggered release will be required (e.g., Eudragit S or enzymatically degradable polysaccharide coatings) together with steric stabilization and mucus-penetration strategies (e.g., cholesterol/PEG-lipids). Given epidemiologic signals at high BC intake in smokers or asbestos-exposed cohorts, dose optimization and patient selection will be essential in vivo.

## Conclusion

Neutral multilamellar liposomes co-encapsulated β-carotene and doxorubicin with high entrapment, nanometric size, and moderately negative ζ-potential. In HCT-116 cells the co-formulation retained doxorubicin’s cytotoxic potency, strengthened G₀/G₁ checkpoint control with marked S-phase depletion, and increased apoptosis, while partially moderating DNA fragmentation relative to DOX-Lipo. Biophysical and spectroscopic data support distinct, complementary drug–bilayer interactions that rationalize the observed mechanism. These in-vitro findings, together with supportive docking and ADMET predictions, establish proof-of-concept for dual-agent delivery and justify stability optimization and in-vivo efficacy/safety studies in colorectal-cancer models.

These in-silico predictions contextualize physicochemical differences; they are not in-vivo PK. Route of administration remains open; if oral colon targeting is pursued, gastric protection (e.g., Eudragit S or enzymatically degradable polysaccharides), steric stabilization (cholesterol/PEG-lipids), and mucus-penetration strategies will be required.

## Data Availability

The datasets generated and/or analyzed during the current study are not publicly available due to restrictions imposed by the institutional review board (IRB) to protect sensitive biological and experimental data, but they are available from the corresponding author upon reasonable request.

## Abbreviations

BC: β-Carotene
DOX: Doxorubicin
BC–DOX: β-Carotene and doxorubicin co-loaded liposomes
CRC: Colorectal cancer
EPR: Enhanced permeability and retention
DLS: Dynamic light scattering
TEM: Transmission electron microscopy
FTIR: Fourier-transform infrared spectroscopy
DSC: Differential scanning calorimetry
PDI: Polydispersity index
EE%: Entrapment efficiency percentage
IC_50_: Half maximal inhibitory concentration
ROS: Reactive oxygen species
UV–Vis: Ultraviolet–visible spectroscopy
SRB: Sulforhodamine B (cytotoxicity assay)
MTT: 3-(4,5-Dimethylthiazol-2-yl)-2,5-diphenyltetrazolium bromide (viability assay, if used)
PBS: Phosphate-buffered saline
ANOVA: Analysis of variance
CI: Combination index
HPLC: High-performance liquid chromatography
BSA: Bovine serum albumin
RPMI: Roswell park memorial institute medium
DAPI: 4′,6-Diamidino-2-phenylindole
DMSO: Dimethyl sulfoxide
µg/mL: Micrograms per milliliter
mV: Millivolt
nm: Nanometer
